# Reducing the effects of radiation damage in cryo-EM using liquid helium temperatures

**DOI:** 10.1073/pnas.2421538122

**Published:** 2025-04-22

**Authors:** Joshua L. Dickerson, Katerina Naydenova, Mathew J. Peet, Hugh Wilson, Biplob Nandy, Greg McMullan, Robert Morrison, Christopher J. Russo

**Affiliations:** ^a^Medical Research Council Laboratory of Molecular Biology, Cambridge CB2 0QH, United Kingdom

**Keywords:** cryo-EM, electron microscopy, radiation damage, water phase transformation, structural biology

## Abstract

The physical limit in determining the atomic structure of biological molecules is radiation damage. In electron cryomicroscopy, there have been numerous attempts to reduce the effects of radiation damage by cooling the specimen beyond liquid-nitrogen temperatures, yet all failed to realize the potential improvement for single-particle structure determination. We have identified the physical causes of information loss at liquid-helium temperatures, and overcome them using a combination of nanoscale electron beam illumination and a gold specimen support with 100 nm diameter holes. This combination allowed structure determination where every frame in the exposure contained more information than was available with cryomicroscopy at liquid-nitrogen temperatures, matching expectations from crystal diffraction. Since a 100 nm hole is smaller than the field of view of a typical micrograph, the edges of the foil are directly visible in each micrograph. Protein molecules that are degraded tend to aggregate at the edges of foil holes and can constitute a significant fraction of the micrograph. This and the need for minimal water-foil irradiation will both be important to consider as new cryomicroscopes and specimen supports are developed for imaging molecules at extremely low temperatures where the effects of radiation damage are reduced.

## Significance

Structure determination by electron cryomicroscopy (cryo-EM) is limited by radiation damage. Even though damage is reduced at liquid-helium temperatures, all attempts solve structures at these temperatures by single-particle cryo-EM have failed to see any benefit. We found this was due to the complex interaction of the electron beam with the specimen, and the surprising fact that amorphous frozen water expands when irradiated at helium temperatures instead of contracting as was previously thought. Using this knowledge, we were able to eliminate the previous problems with imaging at liquid-helium temperatures and obtain structures where every image was better than the equivalent using liquid-nitrogen cooling. Specimen supports with small holes were key to this advance but introduce new challenges to specimen preparation.

It is firmly established that radiation damage is the single most important physical limit in imaging biological molecules (1) because most bonds in the molecule would be broken long before an atomic resolution image of a single molecule could be obtained. Cooling the specimen to liquid-nitrogen temperature (*≃*80 K) is the only successful method to date of reducing the effects of radiation damage in biological electron microscopy (2), and a cryogenic specimen temperature provides the additional benefit of stabilizing aqueous specimens in the vacuum of the electron microscope (3). The major improvements in electron cryomicroscopes in recent years (4) are a direct result of increasing the amount of signal in images before the molecules are destroyed by the beam. Cooling to liquid-helium temperature reduces the effects of radiation damage in crystals by a factor of 5 to 10 relative to room temperature and a factor of about two relative to liquid-nitrogen temperature (5–7). So it is alluring to think that liquid-helium cooling would improve the amount of signal in images by reducing the effects of radiation damage just as it does in diffraction patterns of crystals. Yet independent efforts, spanning several decades, found images taken of liquid-helium cooled specimens were at best no better than liquid-nitrogen (8) and were often worse (9, 10). As a result, electron cryomicroscopy (cryo-EM) at liquid-helium temperature has largely been abandoned. Here, we sought to investigate all factors that deteriorate the data quality in liquid helium cooled cryo-EM, to understand the physics behind them, and to devise methods to eliminate them.

**Fig. 1.**
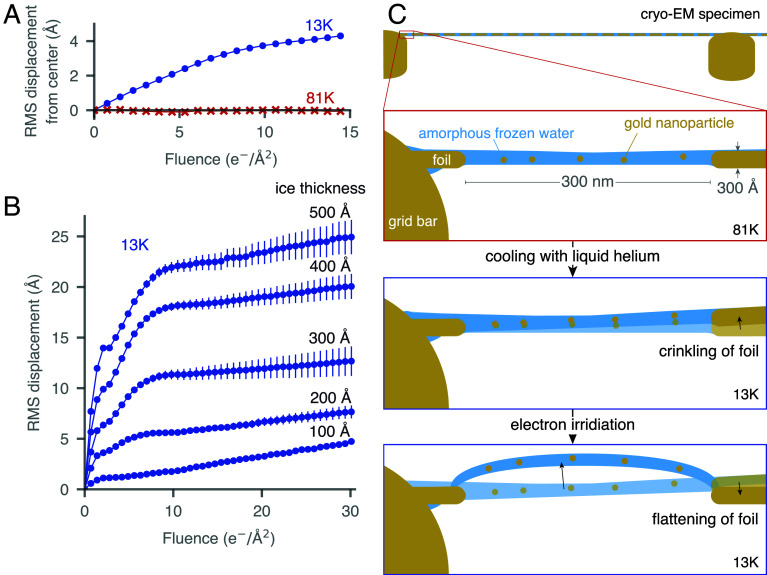
Information loss in cryomicrographs at liquid-helium temperatures is caused by movement of the specimen. The movement of gold nanoparticles in 300 nm diameter holes in an all-gold specimen support was measured using identical imaging conditions at specimen temperatures of 13 K and 81 K (*A*). At 13 K, the particles are moving away from the center; the movement is uncorrelated at 81 K. The movement of a gold specimen support foil during a typical low-dose micrograph at 13 K was measured for a range of frozen water thicknesses [(*B*), error bars represent the SE in the mean]. The cumulative displacement (projected in the image plane at 30° tilt) increases with the thickness of frozen water. We propose a model for the causes of the observed movement (*C*). At 81 K, the gold foil and the amorphous frozen water in the 300 nm diameter holes are flat. On cooling to 13 K, stress accumulates in the foil from differential contraction with frozen water coating its surface, causing it to crinkle. As the specimen is irradiated, the amorphous frozen water becomes an ultraviscous fluid, allowing the foil to relax and flatten. The irradiation also changes the structure of the low-temperature amorphous frozen water, causing it to expand and in some instances buckle.

First, we confirm that movement of the specimen is the primary cause of information loss that led to the abandonment of cryo-EM imaging at liquid-helium temperature (Fig. 1). Unlike in diffraction, where movement does not cause information loss, in imaging, movement of the particles by several Ångstroms in the first few frames of an exposure causes blurring and loss of high-spatial frequency information (8). Even when using small-hole gold grids with 200 to 300 nm diameter holes (11), which eliminate beam-induced motion at liquid-nitrogen temperature (12), we observed that image quality was still reduced at liquid-helium temperature (*SI Appendix*, Fig. S2). To further investigate, we collected data using 300 nm diameter hole gold specimen supports with gold nanoparticles embedded in amorphous frozen water. When the stage was tilted to 30°, the foil exhibited an abrupt motion perpendicular to the tilt axis (Movies S1 and S2), with this motion becoming more pronounced as the frozen water thickness increased (Fig. 1*B*). Further experiments, varying the gold foil thickness, specimen temperature, and beam size (*SI Appendix*, Fig. S3), suggested that this movement was due to the physical motion of the foil itself. Based on these observations, we propose a model where the differential contraction between the gold support foil (13) and the frozen water (14) on its surface, as they are further cooled to liquid-helium temperature, generates stress in the foil. This phenomenon is similar to the “cryocrinkling” observed in carbon foils on copper grid bars (15–17). Upon electron beam irradiation, the frozen water behaves as an ultraviscous fluid (12) allowing the gold foil to relax (Fig. 1*C*).

**Fig. 2.**
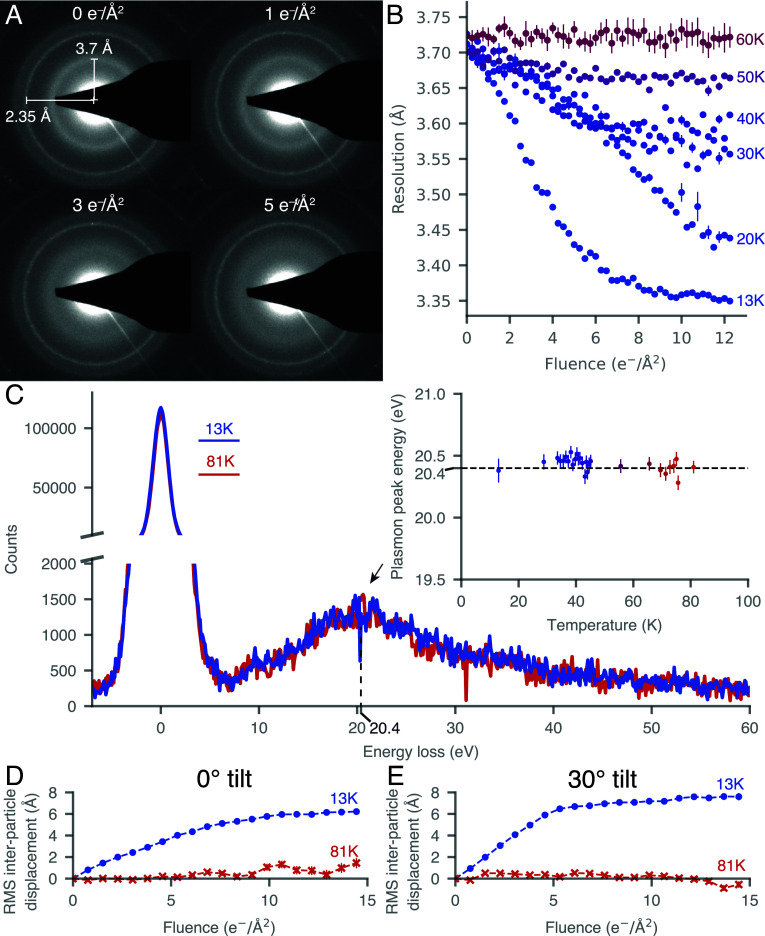
The change in the diffraction pattern of amorphous frozen water during electron irradiation at 13 K is not a high-density phase transformation. In electron diffraction patterns of amorphous frozen water at 13 K, the ring at 3.7 Å became broader, weaker, and shifted to *≈*3.3 Å over a fluence of 5 e^−^/Å^2^ (*A*). The magnitude of the shift was dependent on specimen temperature (*B*) and became unmeasurable above 60 K. Electron energy loss spectra of amorphous frozen water (*C*) showed that the plasmon energy remains at 20.4±0.2 eV between 13 K and 81 K. The plasmon peak energy is plotted versus temperature in the *Inset*, with color as in (*B*) and error bars are the S.E.M. The positions of 5 nm gold nanoparticles in 300 nm diameter holes were measured at 13 K and 81 K with tilt angles of 0° (*D*) and 30° (*E*). These measurements are consistent with observations using X-ray absorption spectroscopy (25).

In addition to the movement of the foil, we observed that gold nanoparticles embedded in amorphous frozen water moved independently of the foil at liquid-helium temperatures (Fig. 1*A*). Surprisingly, this movement was not caused by a collapse of the frozen water into a higher density phase as was previously reported (18, 19). Both of these studies measured a shift in a peak in the diffraction pattern of amorphous frozen water from 3.7 Å to 3.2 Å upon irradiation at liquid-helium temperatures. They interpreted this shift as a phase transition from low-density amorphous (LDA) frozen water [density of 0.93 g/cm^3^ (3)] to a high-density amorphous frozen water [density of 1.1 g/cm^3^ (20)] after 2 e^−^/Å^2^ to 5 e^−^/Å^2^ of irradiation. We have repeated these measurements and confirmed that this peak shift does occur, alongside the peak becoming broader and weaker (Fig. 2*A*). Moreover, we conducted these measurements at specimen temperatures intermediate between liquid-helium and liquid-nitrogen and found that the extent of the peak shift decreases at higher temperatures (Fig. 2*B*), suggesting that the shift reaches a temperature-dependent equilibrium. To further investigate the interpretation of these diffraction patterns as a density change, we sought a method to measure the density of the amorphous frozen water during irradiation in the electron microscope. The resonant energy of the collective oscillations of electrons in water (the plasmon energy) is proportional to the square root of the electron density (21), which is in turn proportional to the water density. This plasmon energy can be measured directly for electrons irradiating amorphous frozen water using electron energy loss spectroscopy (EELS). We measured the position of the water plasmon peak as a function of temperature from 13 K to 81 K (Fig. 2*C*), where each spectrum was collected at a fluence after the diffraction pattern shift had occurred. We found the plasmon peak energy was 20.4±0.2 eV in all instances. Liquid water has a plasmon peak at 21.4 eV (22, 23), whereas the plasmon peak energy is 20.4 eV for LDA frozen water (24). A change in density from 0.93 g/cm^3^ to 1.1 g/cm^3^ would shift the plasmon energy from 20.4 eV to 22.2 eV. Therefore, this measurement excludes the possibility of a change in the density of amorphous frozen water upon irradiation at liquid-helium temperature to a confidence of greater than 5σ.

Another way to determine whether the frozen water collapses into a high-density phase is to measure the position of particles embedded within it during the collapse. If the density increases by almost 20%, the particles will move closer together. We measured the position of gold nanoparticles in frozen water specimens within 300 nm diameter holes upon irradiation at liquid-helium and liquid-nitrogen temperatures. From these positions, we can then calculate the distance between each particle pair as a function of electron fluence (Fig. 2 *D* and *E*). We observed that the root mean squared interparticle distance increased over the first 5 e^−^/Å^2^ by more than 5 Å at 13 K, irrespective of tilt angle. In contrast, we saw no appreciable change in the root mean squared interparticle distance at liquid-nitrogen temperatures. This again excludes the possibility of a collapse to a high-density amorphous water phase. So why are the particles embedded in the frozen water moving apart and why does the peak in the water diffraction pattern shift? We propose that the frozen water is expanding during the first few e^−^/Å^2^ of irradiation at liquid-helium temperatures because the radiolytic fragments of water, which normally recombine or are lost to the vacuum at liquid-nitrogen temperatures, accumulate (25) (*SI Appendix*, Fig. S4). This would explain the shift and broadening of the peak in the diffraction pattern, the absence of the shift in the plasmon peak energy, and the increase in the distance between particles at 13 K compared to 81 K; this is discussed in more detail in *SI Appendix*.

**Fig. 3.**
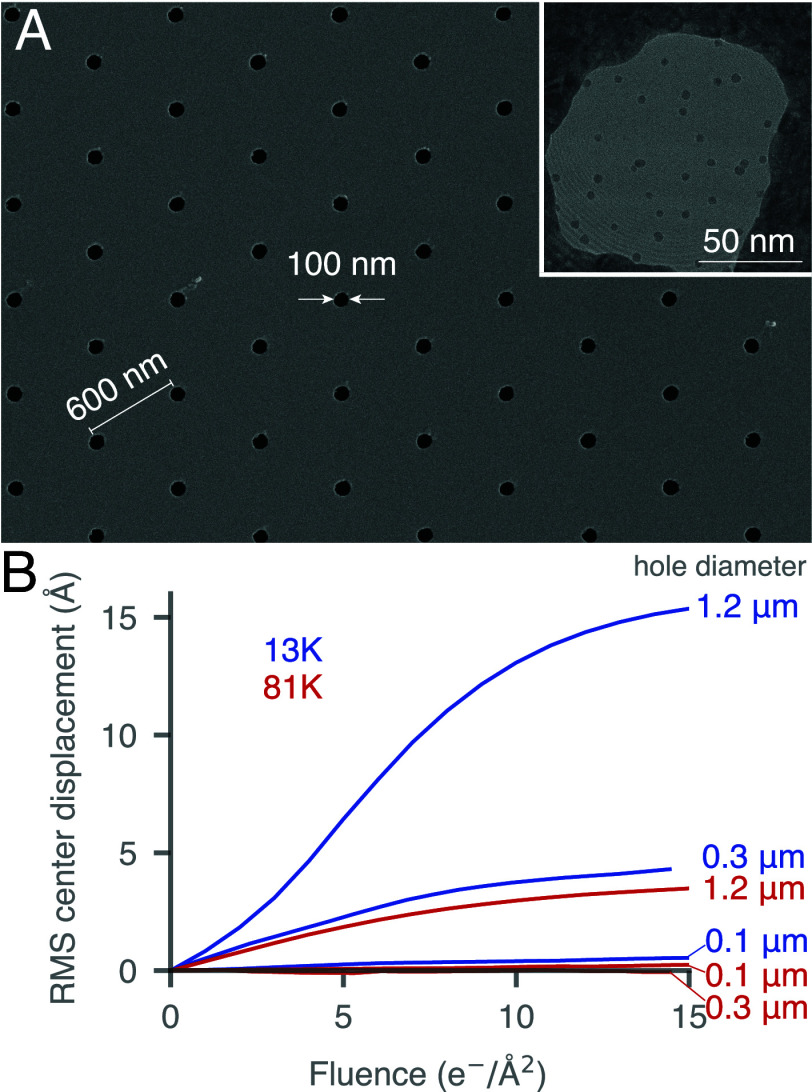
Gold specimen supports with 100 nm diameter holes, combined with a 300 nm diameter electron beam, eliminate specimen movement at 13 K. A specimen support comprising a 300 Å thick gold foil supported on a 3 mm grid, with a hexagonal periodic array of 100 nm diameter holes was fabricated; the surface of which is shown by scanning electron microscopy (*A*). To minimize the movement of the foil during low-dose imaging, the electron beam was limited to being just larger than the hole. The *Inset* shows a transmission electron microscope image of a specimen of 5 nm gold nanoparticles in a 100 nm hole (*SI Appendix*, Fig. S6), where the Fresnel fringes from the small beam are visible across the entire image. Gold nanoparticles were tracked in a series of experiments to compare the movement as a function of hole size and temperature (*B*). For hole sizes above 100 nm, movement was always seen at 13 K even though 300 nm holes were small enough to eliminate movement at 81 K.

The expansion of the amorphous water under irradiation at liquid-helium temperature has profound consequences for the movement of the embedded aqueous specimens. We observed that the movement of the particles was larger in the direction perpendicular to the plane of the specimen (Fig. 2*E* and *SI Appendix*, Fig. S5), which is reminiscent of the buckling of amorphous frozen water previously observed in larger holes at liquid-nitrogen temperatures (12). Frozen water that expands within a foil hole will eventually buckle to release the strain. The amount of expansion required for the buckling to occur depends on how thick the film of frozen water is, the Young’s modulus of the frozen water, and the diameter of the suspended frozen water (7). Since we do not have control over the Young’s modulus of amorphous frozen water and a thin film is needed for single-particle imaging, the most straightforward way to prevent buckling from occurring during electron irradiation at liquid-helium temperatures is to decrease the diameter of the foil holes. Using a method adapted from ref. 11, we made all-gold grids with 100 nm holes. In doing so, we were able to achieve an aspect ratio (hole diameter:foil thickness) of <5:1. By combining these small aspect ratio specimen supports with a 300 nm diameter beam we were able to eliminate movement of the specimen completely (Fig. 3*B*). The small holes eliminated the movement of the frozen water within each hole and the small beam eliminated the movement of the foil by irradiating a minimal area surrounding the hole. The small aspect ratio meant that the differential expansion of the frozen water upon irradiation relative to the contraction of the gold foil never exceeded a threshold for buckling. The small beam was required to minimize the movement of the foil caused by release of stress between the thin film of water on the surface of the foil and the gold (*SI Appendix*, Fig. S3).

**Fig. 4.**
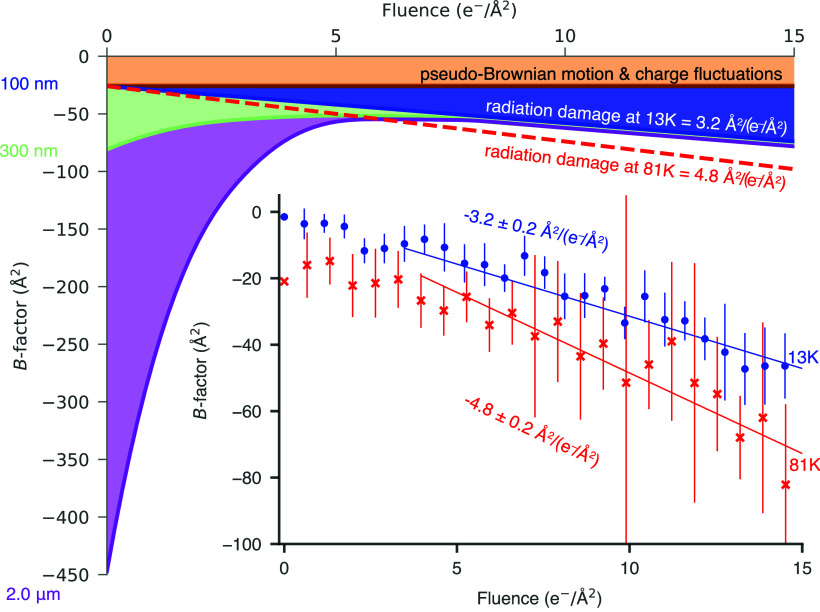
A physical theory for information loss in cryo-EM at liquid-helium temperatures. Through a series of measurements, we isolated independent sources of dose-dependent image blurring at liquid-helium temperatures and used a Gaussian approximation to estimate the amount of atomic displacement they cause in each image. The width of this Gaussian (*B*-factor) characterizes the amount of displacement in each frame, which is analogous to a Debye–Waller factor and can be summed to find the total blurring. At 13 K the blurring is caused by pseudo-Brownian motion and charge fluctuations (constant with dose, brown), radiation damage (blue), and specimen motion (purple: 2 μm holes, green: 300 nm holes, and blue: 100 nm holes). The *Inset* plot shows the measured per-frame *B*-factors in two apoferritin structures determined in 100 nm diameter holes at 13 K and 81 K. The dashed red line thus shows the increase in image blurring caused by radiation damage at 81 K.

To show the improvement in signal for cryo-EM images taken without movement at liquid-helium temperatures using the small hole support and a small beam, we determined six structures: two each of mouse heavy-chain apoferritin at specimen temperatures of 13 K and 81 K (*SI Appendix*, Fig. S6) and one of the DNA protection from starvation protein (DPS) at both 13 K and 81 K (*SI Appendix*, Fig. S8). We performed per-frame reconstructions, which we can use to analyze whether the expected improvement in radiation damage is borne out in single-particle structure determination. Radiation damage is expected to cause the data quality, expressed in terms of a *B*-factor, to decay linearly with dose, as measured by both X-ray and electron crystallography (26–28). This has been demonstrated in single-particle cryo-EM at liquid-nitrogen temperatures using movement-free specimen supports, where the rate of *B*-factor decay was 5 Å^2^/(e^−^/Å^2^). If movement of the specimen at liquid-helium temperatures has also been eliminated, the *B*-factor should decay linearly and at a rate that is 1.2 to 1.8*×* slower than at liquid-nitrogen temperatures; consistent with what has been measured in diffraction studies (7). This first liquid-helium apoferritin dataset decayed at a rate 1.5*×* slower than the corresponding 81 K dataset (Fig. 4) and the second liquid-helium apoferritin dataset (*SI Appendix*, Fig. S7) showed similar improvement. For DPS, we again found a comparable reduction in radiation damage for liquid-helium cooling (*SI Appendix*, Fig. S8), albeit with a slight amount of loss in both datasets at the beginning of irradiation which we attributed to movement of the beam during unblanking that was not related to the specimen temperature. Together these results demonstrate that the radiation damage reduction measured in 2D crystals (7) applies to single-particle cryo-EM and by extension, to cryo-EM of specimens from cells, provided that the specimen does not move.

The Rosenthal-Henderson *B*-factors (29), which measure the number of particles needed to achieve a particular resolution, improved at 13 K compared to 81 K when using 100 nm diameter holes. However, this data quality metric can be strongly influenced by variables that are difficult to control, such as sample quality, ice thickness, and microscope performance. For instance, the aggregation of molecules on the edges of foil holes—a common issue across foils of all sizes—resulted in significantly thicker samples for apoferritin on 100 nm diameter holes than on 300 nm holes. This is likely the primary reason why the Rosenthal-Henderson *B*-factor was worse for these datasets. With specimen motion now eliminated and radiation damage reduced, we investigated whether other physical phenomena could impact data quality, and by extension the Rosenthal-Henderson *B*-factor, at liquid-helium temperatures. Potential factors that could impact data quality include charge accumulation (30–33), pseudo-Brownian motion (34), microscopic charge fluctuations (35), and hydrogen bubbling (24). The conductivity of the support foil is also of concern when lowering the temperature beyond liquid-nitrogen temperatures because the commonly used amorphous carbon is a semiconductor that becomes increasingly insulating at lower temperatures (36). In contrast, the conductivity of gold increases with decreasing temperature; we verified the conductivity of the 300 Å thick foils used for this work at liquid-helium temperatures using a cryogenic four-point probe setup (*SI Appendix*, Fig. S10) and report them in *SI Appendix*, Table S1. Based on our previous theory of dose-dependent information loss at liquid-nitrogen temperatures which included all of these forms of information loss (37), dubbed the “grand scheme,” we remeasured each of these phenomena at 13 K and report the results in *SI Appendix*, Figs. S10–S12. Interestingly, the accumulation of positive charge during electron irradiation is slightly increased at liquid-helium temperatures, probably due to reduced electron-induced conductivity. Regardless, provided the gold support foil is irradiated simultaneously with the amorphous frozen water, charge accumulation saturates within *<*0.1 e^−^/Å^2^ at both 81 K and 13 K, and thus has minimal impact on imaging and the *B*-factor in practical cryo-EM (*SI Appendix*, Fig. S10). Hydrogen bubbling, which could potentially reduce contrast in micrographs (9), occurs more extensively at liquid-helium temperatures compared to liquid-nitrogen temperatures (Movies S3 and S4). But in typical single-particle cryo-EM specimens, this bubbling is not visible until the fluence exceeds 100 e^−^/Å^2^ (Movies S3 and S4), making it of little concern for structure determination or identification since all high-resolution information has been destroyed before then. Additionally, our measurements show that the pseudo-Brownian motion of molecules caused by electron irradiation is the same at both specimen temperatures (*SI Appendix*, Fig. S11), meaning that the *B*-factor contributed by this effect is also the same. Similarly, the impact of microscopic charge fluctuations for frozen water on all gold specimen supports is the same at both temperatures (*SI Appendix*, Fig. S12), meaning that this will contribute negligibly to the overall *B*-factor for each frame.

Thus we now have a theory comprising all known dose-dependent sources of information loss in cryo-EM imaging at liquid-helium temperatures (Fig. 4). We have demonstrated that high-resolution imaging data collected at these temperatures can have an improved signal-to-noise ratio in every frame due to reductions in radiation damage, and the resulting structures have higher resolution at 13 K than at 81 K. This theory presented here offers the opportunity for modifying the specimen preparation and data collection strategy in ways that could eliminate specimen motion with higher throughput. At present, to harness the advantage of reduced damage to the specimen at helium temperatures, care needs to be taken in both specimen preparation and data acquisition to avoid undesired movement during imaging. We found this is achieved by keeping the specimen aspect ratio <5:1, which translates to imaging molecules in 200 Å thick ice in 100 nm holes in a gold foil, and also by keeping the irradiated area below a diameter of 400 nm but still encompassing the entire hole. All the experiments here using liquid-helium cooling were conducted at a specimen temperature of 13 K, which was the minimum practical temperature for this stage design. It is possible that the diffusion of more of the products of radiolysis will be frozen out at temperatures below 13 K, so accurate measurements of radiation damage at temperatures closer to 0 K are needed to look for further potential improvement in radiation damage before investment is made in developing new microscopes at these even lower temperatures. Increasing the signal in each frame of every micrograph will allow smaller proteins to be solved by cryo-EM (for example, the factor of 1.5 increase in critical dose demonstrated here should lower the minimum molecular weight by the same factor, e.g. from 40 kDa to 25 kDa), and also allow smaller molecules to be directly identified by their structure in cells. Equally, it should now be feasible to exploit fully the benefit of reducing the effects of radiation damage with large macromolecular complexes, using the methods described here. Reduced radiation damage using lower temperature electron cryomicroscopy is therefore now poised to help keep the ongoing avalanche of new molecular structures in biology flowing at pace.

## Materials and Methods

All data were collected on a Tecnai Polara G2 electron cryomicroscope (FEI) operated at 300 keV.

### Electron Flux Calibration.

To calibrate the electron beam current, we connected a picoammeter to the drift tube of the Gatan Tridium energy filter attached to the microscope. The beam was focused to a small point and imaged on a TV camera instead of the Gatan US4000 camera to prevent any damage to the camera. The primary energy setting on the filter was changed from 300 keV to 0 keV to measure the current on the picoammeter, and the flu-screen was lowered to measure the current on the flu-screen. Current measurements were taken at spot sizes between 3 and 11, and we ensured that the beam remained on the TV screen at a filter primary energy of 300 keV after each change in spot size. A least squares fit was performed between the screen current and true current measured by the picoammeter, giving a gradient of 1.0473 and intercept of 0.0179 nA.

### Apoferritin Structure Determination.

#### Specimen preparation.

Samples of *Mus musculus* heavy chain apoferritin were produced according to the procedure in ref. 38. After purification, the samples were at a final concentration of 10.8 mg/mL, aliquoted into 20 μL batches and were frozen and stored at −80 ^°^C. HexAuFoil grids (12) with hole diameters of 100, 200, and 300 nm were used for apoferritin specimen preparation. The 100 and 200 nm apoferritin grids were the ones used for structure determination. The grids were exposed to a low-energy plasma (Fischione 1070) comprising a mixture of argon and oxygen (9:1) for 180 s at 70% power (42.0 W forward power, 1.8 W reflected power) to render them hydrophilic. The grids were then passivated immediately before freezing by submerging them in fresh potassium citrate solution (Potassium citrate tribasic monohydrate, Sigma) at 50 mg/mL for 15 min to 30 min. We found this reduced the aggregation of denatured apoferritin particles at the gold–water interface around the edges of the holes. For 300 and 200 nm holes, the specimens were plunge-frozen in liquid ethane at 93 K using a manual plunger designed in-house and a liquid-ethane cryostat (36). These were all in a 4 ^°^C cold room. A sample volume of 3 μL was pipetted onto the foil side of the grid and blotted for 10 s to 15 s from the same side by Whatman No. 1 filter paper. For 100 nm holes, specimens were plunge-frozen in an FEI Vitrobot equilibrated at 4 ^°^C and 100% relative humidity. A sample volume of 3 μL was pipetted onto the foil side of the grid and the grid was manually blotted in the Vitrobot chamber for 30 s from the grid bar side by Whatman No. 1 filter paper, before being blotted from both sides for 60 s. This procedure helped create thinner layers of specimen in the very small holes. All samples were stored in liquid nitrogen until imaging in the electron microscope.

#### Data collection.

Data were collected manually on a transmission electron microscope (TEM) (FEI Polara G2 TEM) operating at an accelerating voltage of 300 kV and cooled using either liquid nitrogen or liquid helium in the inner dewar. The stage temperature was measured using a silicon diode near the specimen (reading the temperature of the surrounding cryobox in thermal contact with the cartridge) and was 81 K when cooling with liquid nitrogen and 12 K to 15 K when cooling with liquid helium. On delivery of the microscope in 2006, the cryobox temperature was compared to a cartridge mounted with a silicon diode. When cooled with liquid helium, the cartridge diode read 13.679 K and the silicon diode mounted to the cryobox read 15.083 K. Cooling the microscope to 13 K requires around 15 L of liquid helium, which allows 6 h of data collection and, at the time of these experiments, cost around £400 to £500 per experiment. To reduce the impact of airflow on data quality, an aluminum enclosure was built around the microscope (*SI Appendix*, Fig. S1). Acoustic noise was reduced from *≈*60 dB to *<*40 dB by removing all computers and processors from the room and lining the enclosure with soundproof foam. Magnetic fields were reduced to *<*0.1 mGauss at the column by deflecting some AC fields generated nearby with mu-metal. Together, these modifications to the Polara room brought the information limit of the microscope from less than 4 Å to better than 1.8 Å in all directions (*SI Appendix*, Fig. S1 *B* and *C*).

For 200 nm hole data collection, the microscope was set at a nominal magnification of 59,000*×*, and the camera was a K2 direct electron detector (Gatan) operating in counting mode. The C2 intensity was set at 56.9%, which is the parallel condition, as determined using the diffraction of the gold foil and following the procedure outlined in ref. 39. A 30 μm diameter C2 aperture was used with a 100 μm diameter objective aperture. The beam size was 1 μm, measured by lowering the magnification in imaging mode and comparing the beam diameter to the distance between the holes. The flux was calculated using the current reading on the flu-screen, which was calibrated using a picoammeter as described above. The flux was measured as 12.7 e^−^/Å^2^/s (0.63 e^−^/Å^2^/frame), with an exposure time of 5 s, to give a total fluence of 63.5 e^−^/Å^2^. When collecting data, movies were collected on every other hole to avoid collecting on preirradiated regions, and there was a 30 s wait after each stage move to reduce stage drift during the exposure.

For the 100 nm diameter holes, the microscope was set at a nominal magnification of 93,000*×* and a 10 μm diameter C2 aperture was used to give a beam size in the parallel condition of 300 nm. The flux for the first dataset (13 K) was 10 e^−^/Å^2^/s and exposure time 5 s. In the second dataset, the liquid-nitrogen cooled data were collected at a flux of 17 e^−^/Å^2^/s, with a total exposure of 3 s. The liquid-helium cooled dataset was collected at a flux of 12.4 e^−^/Å^2^/s, with a total exposure of 4 s. The data collection parameters are summarized in *SI Appendix*, Table S3.

#### Data processing.

The pixel size was calibrated by using the gold lattice present in all of the images and the program magCalEM (40). This was determined to be 0.648 Å for the 200 nm hole datasets and 0.415 Å for the 100 nm hole datasets. To process the data, the tiff movies were imported into RELION 4.0 (41) and motion corrected with RELION’s own implementation of MotionCor2 (42). CTF estimation was performed with CTFFIND-4.1.13 (43). Particles were picked using crYOLO (44) and extracted into 400 *×* 400 pixel boxes. For the 100 nm hole datasets, the particles were rescaled to a pixel size of 0.83 Å. Cycles of 3D autorefinement with octahedral symmetry, CTF refinement (45), and Bayesian polishing (46) were performed until the resolution converged. Cryosieve (47) was used along with cycle of 3D classification without alignment to remove bad particles. Reconstructions from subsets of particles were used for estimation of the Rosenthal *B*-factor (29). Per-frame reconstructions were made from the first 30 frames using RELION movie reconstruct, and the ratios of amplitudes were compared to the first frame were calculated according to Eq. **1** (48):

[1] $$\begin{array}{c} \frac{\tau_{f}\left(q\right)}{\tau_{1}\left(q\right)}=\sqrt{\frac{FSC_{f}\left(q\right)-FSC_{f}\left(q\right)FSC_{1}\left(q\right)}{FSC_{1}\left(q\right)-FSC_{f}\left(q\right)FSC_{1}\left(q\right)}}, \end{array}$$

where the subscripts *f* and 1 denote the *f*th frame and first frame respectively, *q* is the spatial frequency, *τ* is the amplitude, and *FSC* is the Fourier shell correlation between the two data half-sets.

### DPS Structure Determination.

#### Specimen preparation.

The DPS (DNA protection from starvation enzyme) was produced as previously described (49). HexAuFoil grids with hole diameters of 100 nm were used. The grids were exposed to a low-energy plasma (Fischione 1070) comprising a mixture of argon and oxygen (9:1) for 180 s at 70% power (42.0 W forward power, 1.8 W reflected power) to render them hydrophilic. The grids were then passivated immediately before freezing by submerging them in fresh potassium citrate solution (Potassium citrate tribasic monohydrate, Sigma) at 50 mg/mL for 15 min to 30 min. An aliquot stored at −80 °C was diluted to 1.2 mg/mL and a volume of 3 μL was pipetted onto the foil side of the grid and blotted in a 4 °C cold room for 30 s from the same side by Whatman No. 1 filter paper.

#### Data collection.

Data were collected manually on a TEM (FEI Polara G2 TEM) operating at an accelerating voltage of 300 kV and cooled using either liquid nitrogen or liquid helium in the inner dewar. The camera was a Gatan K2 operating in counting mode. The microscope was set at a nominal magnification of 93,000*×* and a 10 μm diameter C2 aperture was used to give a beam size in the parallel condition of 300 nm. The flux for the liquid-nitrogen cooled dataset was 11.0 e^−^/Å^2^/s and exposure time 5 s. The flux for the liquid-helium cooled dataset was 9.7 e^−^/Å^2^/s and exposure time 6 s. The data collection parameters are summarized in *SI Appendix*, Table S3.

#### Data processing.

The data processing was performed in the same way as for apoferritin, but tetrahedral symmetry was applied and the particles were rescaled to a pixel size of 0.92 Å.

### Gold Nanoparticle Tracking Experiments.

#### Specimen preparation.

HexAuFoil R0.3/0.3, HexAuFoil R0.1/0.5, and UltrAuFoil R1.2/1.3 grids were rendered hydrophilic by exposure to a low-energy plasma (Fischione 1070) comprising a mixture of argon and oxygen (9:1) for 90 s. For HexAuFoil grids, 5 nm diameter citrate functionalized gold nanoparticles (Nanocomposix) were concentrated from 1 mg/mL to 5 mg/mL by centrifugation for 45 min at 20,238 rcf. For UltrAuFoil grids, 10 nm diameter gold nanoparticles (BBI) at OD 100 were used. A 3 μL solution of nanoparticles was pipetted onto the foil side of the grids and blotted for 10 s to 15 s from the same side using Whatman No. 1 filter paper. The specimens were plunge-frozen in a cold room using a manual plunger designed in-house and a liquid-ethane cryostat (36) set to 93 K and stored under liquid nitrogen at 77 K before imaging.

#### Data collection and processing.

The samples were imaged on an FEI Polara G2 TEM operating at an accelerating voltage of 300 kV. Movies were collected at specimen temperatures of 13 K and 81 K, and also at stage tilts of 0° and 30°. For the HexAuFoil datasets, the camera was a Gatan K2 operating in counting mode. For 300 nm diameter holes, the magnification was 59,000*×*, the beam diameter was 1.2 μm, the flux 0.7 e^*−*^/Å^2^/frame (20 frames per second) and total fluence 60 e^*−*^/Å^2^. For 100 nm diameter holes, the magnification was 93,000*×*, the beam diameter was 0.3 μm, the flux 10.2 e^*−*^/Å^2^/s (1.0 e^*−*^/Å^2^/frame) and total exposure 2.5 s. For the UltrAuFoil data, the camera was a Falcon3 operating in linear mode, the beam diameter was 1.8 μm, the magnification was 31,000*×*, the flux 1.0 e^*−*^/Å^2^/frame, and the total fluence 62 e^*−*^/Å^2^. Movies (*≈*100 for each hole size and tilt angle) were aligned using Unblur (50), and gold nanoparticles not bound to others or the foil were selected using custom Python scripts. Individual particle movies were then extracted from the aligned movies and motion-corrected again with Unblur. For the HexAuFoil R0.3/0.3 dataset, the thickness of the frozen water in each hole was estimated using the method outlined in *Frozen Water Thickness Estimation*. Custom Python scripts were written to calculate the metrics plotted in *SI Appendix*, Fig. S5 and Figs. 1*B* and 3*B*.

### Frozen Water Thickness Estimation.

To measure the thickness of the water layer in each hole, approximately every hour the stage was moved to an empty area and an image was taken. The counts in each pixel for both blank images and images of frozen water were divided by the fractional loss, *l*, to correct for coincidence loss and imperfect DQE (51),

[2] $$\begin{array}{c} l=DQE\times e^{-\frac{\mathrm{Ds}}{F}}, \end{array}$$

where *D* is the flux density in e^−^/px/s, *F* the frame rate of the camera (400 frames/s), *s* is a fitted parameter (5.149 px^2^), and the DQE is 86.56%. The blank images were sorted by time and each frozen water image was assigned an incident beam intensity based on linear interpolation.

To measure the frozen water thickness, a modified version of the program MeasureIce (52) was used, which is based on the aperture-limited scanning method described in ref. 53. The program uses multislice simulations to calculate the electrons scattered outside of an objective aperture by both elastic and inelastic scattering. To use this method, the size of the objective aperture in frequency space must be known. This was measured by taking gold diffraction patterns and comparing the edge of the objective aperture to the gold (111) ring at 2.35 Å. The edge of the 100 μm objective was measured to be at 1.2 Å resolution (*SI Appendix*, Fig. S13).

MeasureIce was then modified in several ways. First, a command line interface was created to allow it to process hundreds of images without user intervention. Second, the calculation of inelastic scattering was modified to improve its accuracy. The program used an average energy loss for inelastic scattering, taken from ref. 54. Instead of using one value, an energy loss distribution taken from an experimental EEL spectrum (Fig. 2) and calculated the scattering distribution for each 1 eV of energy loss was used. Additionally, the MeasureIce calculated Lorentzian distribution of inelastic scattering was modified to follow the method used in ref. 55. MeasureIce outputs thickness “maps,” which are images where each pixel corresponds to the thickness of that pixel in nm. To measure the average frozen water thickness of the hole, the images were thresholded to remove the gold foil and gold nanoparticles using custom Python scripts and the average value of the remaining pixels was calculated.

### Foil Movement Experiments.

#### Preparing thick foil grids.

HexAuFoil R0.3/0.3 with a foil thickness of 30 nm were loaded into a custom electron beam evaporator (Moorfield) and half of each grid was covered with a mica sheet. The stage was cooled using liquid nitrogen to a temperature of 84 K to 92 K. 99.999% pure gold (Kurt J. Lesker) was deposited onto the foil side of the grids by 9 kV electron beam evaporation at a rate of *≈*1 Å/s, as measured by a calibrated crystal thickness monitor (Infinicon). A total of 35 nm of gold was deposited, resulting in grids with half 30 nm thick foil and half 65 nm thick foil. Apoferritin was applied and frozen as described previously for 300 nm hole diameter grids.

#### Data collection.

In all cases, data were collected on R0.3/0.3 HexAuFoil grids at a magnification of 59,000*×* and with the stage tilted to 30°, which was verified by taking images at a magnification of 9,600*×* and fitting ellipses to the holes in the foil using a custom Python script. All movies were collected on a Gatan K2 operating in counting mode, and each consisted of 100 frames at a flux of 0.4 e^*−*^/Å^2^/frame. Data were collected on the specimens of gold nanoparticles described previously to measure foil motion as a function of frozen water thickness. Data were also collected on apoferritin specimens to measure the foil movement as a function of temperature, beam size, and foil thickness. For the temperature series, data were collected with a 30 μm diameter C2 aperture and a parallel beam, giving a beam diameter of 1 μm. Ten movies were collected at a specimen temperature of 13 K, then ten movies were collected every 5 K as the microscope warmed up from 20 K to 60 K. For the beam size series, the same parameters were used, but with a specimen temperature of 13 K and C2 apertures of 10, 50, and 150 μm giving beam diameters of 0.4, 2.0, and 6.0 μm, respectively. Fifteen movies were collected for each beam size. For the foil thickness series, the C2 aperture was 50 μm (beam size 2.0 μm) and the specimen temperature was 13 K. 68 movies were collected on the 65 nm thick foil half of the grid and 60 movies on the 30 nm thick foil half.

#### Data analysis.

Every movie was aligned using Unblur (50) and the per-frame displacement perpendicular to the tilt axis relative to the first frame was calculated (*SI Appendix*, Fig. S3). Custom Python scripts were used to plot the cumulative displacement along this direction.

### Phase Change Experiments.

#### Specimen preparation.

Specimens were either pure amorphous frozen water or 30% hydrogen peroxide (Sigma) on R0.3/0.3 HexAuFoil grids or R1.2/1.3 UltrAuFoil grids. For the diffraction data and EELS data at 13 K and 81 K, 3 μL of the solution was pipetted onto the foil side of the HexAuFoil grids and blotted for 5 s to 10 s from the same side by Whatman No. 1 filter paper. For the intermediate temperature EELS data, 3 μL of water was pipetted onto the foil side of the UltrAuFoil grids and blotted for 15 s from the same side by Whatman No. 1 filter paper. The specimens were plunge-frozen in a cold room using a manual plunger designed in-house and a liquid-ethane cryostat (36) set to 93 K and stored under liquid nitrogen at 77 K before imaging.

#### Diffraction data collection and analysis.

Diffraction data were collected on an FEI Polara G2 TEM operating at an accelerating voltage of 300 kV and cooled using liquid helium. The beam size was 1.8 μm, ensuring that the gold (111) ring could be used to calibrate the camera length, which was set to a nominal value of 470 mm. The camera was an Orius SC100 CCD, and the flux and exposure time were set such that there was 0.25 e^*−*^/Å^2^/frame. The total fluence was 12.25 e^*−*^/Å^2^ (50 frames). Ten data series were collected at a specimen temperature of 13 K for both pure water and peroxide specimens. To determine the position of the first ring in the diffraction pattern, the centers of the diffraction patterns were identified with BACKAUTO (56), and Python scripts were used to calculate the radial profile by excluding pixels in the bottom 20th percentile to eliminate the beam stop. Subsequently, the gold (111) and frozen amorphous water peaks were fitted using a combination of Voigt and linear profiles via the lmfit library (57).

#### EELS data collection and analysis.

The EELS data were all collected on specimens of pure amorphous frozen water. EELS data were collected on an FEI Polara G2 TEM operating at an accelerating voltage of 300 kV and specimen temperatures between 13 K and 81 K. The 13 K and 81 K datasets were collected in a separate session to the intermediate temperatures. The camera was a Gatan US4000 after a Gatan Tridiem 864 imaging spectrometer, and the dispersion was set to 0.1 eV per pixel, which was verified using the energy loss of aluminum. Two data collection modes were set up: a high-dose mode at 90 e^*−*^/Å^2^/s that was used to preirradiate the sample for 3 s, and a low-dose mode with a flux density *>*100*×* lower to collect the spectra with 0.1 s exposures. Nine spectra were collected at 13 K and eight at 81 K. The data for intermediate temperatures were 0.5 s exposures at a flux of 23 e^*−*^/Å^2^/s. Custom Python scripts were used to fit Voigt profiles to the zero-loss peak (first approximated as the maximum) and the plasmon peak (first approximated to be at 21 eV) to refine the positions of the centers.

### Fabrication of 100 nm Hole Diameter Grids.

TEM grids with 100 nm holes were fabricated following a modified version of the process of Naydenova and Russo (11). In brief, first a 4-inch silicon (100) wafer was patterned with a hexagonal array of wells (100 nm diameter, 600 nm spacing) by Talbot lithography and a Bosh reactive ion etch (Eulitha). Next, the wafer was cleaned with piranha (1 min, 3:1 sulfuric acid:hydrogen peroxide [Sigma]) and a thin layer of copper (50 Å) and then subsequently gold (300 Å) were deposited at a rate of *≈*1 Å/s by electron beam evaporation from high-purity pellets (Kurt J. Lesker, copper: EVMCU50QXQJ and gold: EVMAU50QXQ). The deposition was in a custom 9 kV electron beam evaporator (Moorfield, Minilab 080), evacuated to $5\times 10^{-8}\text{mbar}$, and equipped with a liquid nitrogen cooled stage and two crystal thickness monitors (Inficon). The wafer was held in a custom holder in contact with the cooled stage and kept at 80 K to 90 K during deposition. After deposition, the wafer was allowed to warm to ambient temperature under vacuum over 10 h to 15 h.

To define the individual TEM grids, 3 mm discs were patterned on the wafer using photolithography. A *≈*1 μm layer of resist (Megaposit SPR220-3) was spun onto the wafer (6 krpm, 60 s), which was then soft-baked (99 °C, 90 s), and exposed on a mask aligner (Quintel) through a custom chrome-on-quartz mask (Compugraphics). The wafer was then soft-baked again (99 °C, 120 s), and developed (Microposit MF319, 2 min 15 s) before being hard-baked (85 °C, 10 min), and etched (Transene, TFA gold etch, 40 s). Finally, the resist was removed (Microposit 1165) and the wafer was rinsed with acetone followed by isopropanol and water (MilliQ).

A second round of lithography defined grid bars and rims. An 8 μm to 10 μm layer of resist (Megaposit SPR220-7) was spun onto the wafer (2 krpm, 60 s). The wafer was soft-baked (99 °C, 180 s), exposed through a second mask (35 s), and then incubated at room temperature in the dark for 100 min. Following this the wafer was soft-baked (99 °C, 135 s), developed (6 mins MF26A), and hard-baked (85 °C, 22 min). Finally, an 8 μm to 10 μm layer of gold was electroplated onto the wafer at 54 °C using a 4-inch wafer electroplating bath (Yamamoto) and gold-plating solution (Metalor Technologies SA, MetGold ECF 33B).

As the last step, the grids were released from the wafer by immersion of the wafer in an aqueous solution of 30% KOH (Sigma), 4% polydiallyldimethylammonium chloride (Sigma, 409014) at 80 °C. The released grids were then incubated in 30% KOH at 80 °C for 10 min, washed with deionized water twice, incubated for 5 min in room temperature piranha etch, and then washed with water three times. The grids were tipped out onto filter paper and left to dry.

Before plunge-freezing, grids were placed in FeCl_3_ (Sigma) for 2 h, since this was found to improve particle distribution in the holes. The grids were then washed through a series of 6 washes: 20% HCl, 2% HCl, 0.2% HCl, and 3 washes in 18 MΩ deionized water (Millipore), before being tipped onto filter paper and left to dry.

### Conductivity Measurements.

We measured the resistivity of gold and carbon foils using a cryo-four-point probe instrument (*SI Appendix*, Fig. S9*A*) similar to that in ref. 58. It comprised four gold spring pins 3 mm apart that press onto the specimen, held in place with two sheets of Macor. To prepare the gold specimen, 99.999% pure gold (Alfa Aesar) was deposited onto freshly cleaved mica sheets (Agar Scientific) to a final thickness of 30 nm using the method described previously. The mica was cut into a 15 mm *×* 8 mm rectangle and clamped into the four-point probe instrument. A source measurement unit (Keithley 2450) was used to source current between *−*20 and *+*20 mA in 2 mA steps and measure voltage. Current-voltage (I-V) characteristics (*SI Appendix*, Fig. S9*B*) were measured at room temperature as well as 77 K and 4 K by submerging the apparatus in liquid nitrogen and liquid helium respectively. These measurements are then converted to a sheet resistance, *R*_*s*_,

[3] $$\begin{array}{c} R_{s}=\frac{\pi }{\ln2}\frac{\mathrm{dV}}{\mathrm{dI}}C_{1}C_{2}, \end{array}$$

where $\frac{\mathrm{dV}}{\mathrm{dI}}$ is the gradient of the voltage, *V*, versus current, *I*, and $C_{1}$ and $C_{2}$ are geometric correction factors (59). For the gold foil specimen, $C_{1}=0.5192$ and $C_{2}=1$. The sheet resistance is then converted to bulk resistance by multiplying by the specimen thickness.

For the carbon specimen, amorphous carbon foils of 50 nm thickness were created by vacuum deposition from pulsed heating of carbon threads onto freshly cleaved mica (Agar Scientific) in a high vacuum chamber (Leica EM ACE600). The system was pumped to a pressure of $2.5\times 10^{-6}$ mbar, and the continuously rotating stage was at a distance of 50 mm. The carbon foil on mica was then cut to a 14 mm *×* 6 mm rectangle, and the voltage was measured at room temperature between a current of *−*20 and *+*20 μA in 2 μA steps. At 77 K, the current sourced was *−*200 nA to *+*200 nA in 20 nA steps, and at 4 K, no reliable reading could be taken. To circumvent this, the resistance of a 3 mm diameter carbon rod (Agar Scientific) cut to a length of 13 mm was also measured at the three temperatures. The current sourced was *−*200 mA to *+*200 mA in 20 mA steps.

### Charge Accumulation.

Data were collected on 300 nm hole diameter HexAuFoil grids of apoferritin described above at specimen temperatures of 81 K and 13 K. The magnification was 2,150*×*, which is in LM mode and hence allows the use of the first intermediate lens at very high underfocus, in this case 5 mm. The flux was 0.07 e^*−*^/Å^2^/s. Movies were collected in each selected area with an exposure time of 0.025 s/frame and final exposure of 1.4 s, giving a total fluence of 0.1 e^*−*^/Å^2^. Eleven areas were collected at 13 K, and six areas at 81 K. The beam area relative to the first frame was measured for each frame using a custom Python script and averaged over the dataset.

### Charge Fluctuations.

The specimens for the charge fluctuation measurements were 5 nm diameter gold nanoparticles on HexAuFoil R0.3/0.3 grids, as described above. Data were collected on an FEI Polara G2 TEM operating at an accelerating voltage of 300 kV and specimen temperatures of 13 K and 81 K. The magnification was 59,000*×* (pixel size 0.648 Å), the flux was 10 e^*−*^/Å^2^/s, and exposure time 1 s. 40 frames were collected for each movie on a Gatan K2 operating in counting mode. A series of movies were collected on each hole, at defocus ranges of *−*10–0 μm in 1 μm steps. After collecting a movie series, the electron beam was left irradiating the sample until all of the frozen water was removed. Another movie series was then collected over the same defocus range. A total of 35 holes were imaged at 81 K and 27 at 13 K.

To analyze the data, the defocus was estimated for each movie using CTFFIND-4.1.13 (43) and the movies were aligned using Unblur (50). Custom Python scripts (60) were used to select particles and regions of the foil that diffracted strongly throughout the movie series. The power in the gold-111 diffraction spot was measured as a function of defocus. The fading of the power with increasing defocus was fitted to the spatial coherence envelope function by least-squares fitting, with the following function: (61, 62)

[4] $$\begin{array}{c} E_{s}\left(q\right)=\exp\left[-{\left(\frac{\pi \alpha }{\lambda }\right)}^{2}{\left(\lambda \Delta zq+C_{s}\lambda^{3}q^{3}\right)}^{2}\right], \end{array}$$

where $E_{s}\left(q\right)$ is the fractional amplitude at frequency *q*, and *α* is the semiangle of the source electron distribution, defined as the value where it reduces to 1/e of its value at the origin. The fitted values of the semiangle were then averaged over each dataset for the individual particles, the foil with frozen water, and the foil without frozen water.

### Brownian Motion.

Data were collected on the gold nanoparticle specimens on R0.3/0.3 HexAuFoil grids described previously. Data were collected on an FEI Polara G2 TEM operating at an accelerating voltage of 300 kV and specimen temperatures of 13 K and 81 K. The magnification was 78,000*×* (pixel size 0.495 Å), the flux was 7.5 e^*−*^/Å^2^/s, and exposure time 10 s. Movies of 200 frames each were collected on a Gatan K2 operating in counting mode. A total of 75 movies were collected at 13 K and 87 at 81 K.

The movies were gain-corrected and aligned using Unblur (50) and the gold foil was cropped out of the images. Gold nanoparticles were removed using the program *fidder* (63) and replaced with random noise from a Gaussian distribution of mean equal to that of the cropped image. Frames of the movies were then grouped and summed in batches of *m* frames, where *m* is 1, 2, 4, 5, 8, 10, 20, 25, 50, 100, and 200, to make new movies with different fluences per frame. Power spectra were taken for each frame in these summed movies and the resulting M/m power spectra, where *M* is the total number of frames, were summed so that there is one summed power spectrum for each movie at each frame grouping.

The power spectra were radially averaged and the peak at 3.7 Å (81 K) or 3.3 Å (13 K) was fitted to a Gaussian profile *+* linear fit using custom Python scripts. The linear fit was taken as the background and the area in the Gaussian fit above this line was taken as the peak intensity. The peak intensity was averaged over all movies of the same fluence per frame for each temperature. The peak intensity was plotted against fluence per frame and the latter linear part of the curve was fitted to a straight line. This linear component was subtracted from the peak intensity values to remove any existing correlation between frames, such as an imperfect gain correction. The residual peak intensity values were fit to the following equation: (34)

[5] $$\begin{array}{c} W_{M,m}\left(u\right)\propto \frac{2Md}{\alpha_{u}}\left(\alpha_{u}md+\;\text{exp}\left(-\alpha_{u}md\right)-1\right)/\alpha_{u}md, \end{array}$$

where *M* is the total number of frames, *d* the fluence per frame, $W_{M,m}$ the peak intensity for the sum of M/m power spectra at a spatial frequency *u*, and *α*_*u*_ is as follows:

[6] $$\begin{array}{c} \alpha_{u}=2\pi^{2}\sigma_{0}^{2}u^{2}, \end{array}$$

where $\sigma_{0}^{2}$ is the mean-squared movement in a given direction per e^*−*^/Å^2^.

### Hydrogen Bubbling.

R2/2 UltrAuFoil grids were exposed to a low-energy plasma (Fischione 1070) comprising a mixture of argon and oxygen (9:1) for 90 s at 70% power (42.0 W forward power, 1.8 W reflected power) to render them hydrophilic. 3 μL of *Mus musculus* heavy chain apoferritin at a concentration of 10.8 mg/mL were pipetted onto the foil side of the grid and the grid was manually blotted for 10 s to 15 s from the same side by Whatman No. 1 filter paper. The specimens were plunge-frozen in a cold room using a manual plunger designed in-house and a liquid-ethane cryostat (36) set to 93 K and stored under liquid nitrogen at 77 K before imaging.

Data were collected on an FEI Polara G2 TEM operating at an accelerating voltage of 300 kV and specimen temperatures of 13 K and 81 K. The magnification was 12,000*×* (pixel size 3.04 Å), the flux was 25 e^*−*^/Å^2^/s, and exposure time 2 s. The camera was a Gatan K2 direct electron detector operating in linear mode. A total of 19 image series were collected at each temperature, with each lasting until the hole had popped.

## Supplementary Material

Appendix 01 (PDF)

Movie S1. **59,000× magnification image of the gold foil movement at 13K.** The grid is a 300 nm hole diameter HexAuFoil grid with apoferritin. The sample was irradiated with 300 keV electrons at a flux of 0.4 e^−^/Å^2^/frame. The motion of the image is perpendicular to the tilt axis.

Movie S2. **31,000× magnification image of the gold foil movement at 13K.** The grid is a 300 nm hole diameter HexAuFoil grid with apoferritin. The sample was irradiated by 300 keV electrons at a flux of 0.4 e^−^/Å^2^/frame. The motion of the image is perpendicular to the tilt axis.

Movie S3. **Bubbling of apoferritin specimens at temperatures of 81K and 13K**. The grid is an R1.2/1.3 UltrAuFoil grid and the specimen apoferritin. The sample is irradiated by 300 keV electrons at a flux of 50 e^−^/Å^2^/frame. Hydrogen bubbling is significantly more abundant at the lower temperature but is still only visible after a fluence of > 100 e^−^/Å^2^.

Movie S4. **Bubbling of Hepetitis B viral capsid specimens at temperatures of 81K and 13K**. The grid is an R1.2/1.3 UltrAuFoil grid and the specimen the capsid from the Hepatitis B virus. The sample is irradiated by 300 keV electrons at a flux of 64 e^−^/Å^2^/frame. Hydrogen bubbling is significantly more abundant at the lower temperature and is still only visible after a fluence of > 100 e^−^/Å^2^ after the high resolution features of the molecules are lost. In addition, the bubbles are clearly associated with the individual capsids at 13K whereas they are only roughly correlated with their positions at 81K. Further work is needed to determine if the hydrogen gas that is generated is from the protein, the water or both.

## Data Availability

The final reconstructed maps, half maps, and masks for the six structures (three each at 13 K and 81 K) are deposited in the Electron Microscopy Data Bank (EMD 51777-51782) (64–71). The entire datasets and the per-frame reconstructions for each structure are publicly available in the Electron Microscopy Public Image Archive (EMPIAR-12341) (72).
