# Supplementary material for: Reducing the effects of radiation damage in cryo-EM using liquid helium temperatures
Source: Proc Natl Acad Sci U S A. 2025 Apr 22;122(17):e2421538122. doi: 10.1073/pnas.2421538122 (PMC12054821; doi:10.1073/pnas.2421538122)
Supplement: Supplementary file 1 — Appendix 01 (PDF) [file pnas.2421538122.sapp.pdf]

Supporting Information for

Reducing the effects of radiation damage in cryo-EM using liquid helium temperatures

Joshua L. Dickerson, Katerina Naydenova, Mathew J. Peet, Hugh Wilson, Biplob Nandy, Greg McMullan, Robert Morrison, & Christopher J. Russo

Christopher J. Russo
E-mail: crusso@mrc-lmb.cam.ac.uk

This PDF file includes:

Figs. S1 to S13
Tables S1 to S3
SI References

Supplementary Text

The grand scheme revisited: a physical theory of information loss in cryo-EM at liquid-helium temperatures.

Recent work has led to a physical theory of the dose dependent information loss in cryo-EM at liquid-nitrogen temperature developed by Russo and Henderson (1). It was originally was called the "Rough Grand Scheme" as it encompassed all known physical forms of information loss in cryo-EM specimen imaging, but accurate measurements of the various phenomenon thought to be contributing, were not yet accurately measured. This theory encompasses several critical factors: the radiation damage limit, pseudo-Brownian motion of water, microscopic charge fluctuations, charge accumulation, and beam-induced specimen motion. With the advent of all-gold specimen supports (2, 3) and the use of smaller hole sizes (4), the impacts of charge accumulation and beam-induced specimen motion are now negligible at liquid-nitrogen temperature and the limit set by radiation damage and pseudo-brownian movement described in the grand scheme was reached. Here, we detail our measurements to determine these physical limits at liquid-helium temperatures to look for any possible additional sources of information loss and thus understand if there are benefits to imaging at lower temperatures.

Radiation damage.

Ionizing damage from the primary electron beam as well as from subsequently produced secondary electrons and reactive species impacts the protein molecules during imaging. This can be modelled globally as a rate of change in the B-factor, which has been measured at 5 $Å^2/(e^-/Å^2)$ for single-particle cryo-EM with a primary beam energy of 300 keV and a specimen cooled to liquid-nitrogen temperature. We performed per-frame reconstructions of apoferritin and fitted a B-factor to the amplitudes between 10–4 Å resolution for the 100 nm hole datasets and 9–3 Å for the 200 nm hole datasets(5). The B-factor at a fluence of 0 $e^-/Å^2$ was set as the y-intercept of this fit. The resolution range was chosen to be higher for the 200 nm dataset since the resolution of the per-frame maps was also higher. We measured values of 5.2 ± 0.3 $Å^2(/e^-/Å^2)$ (Fig. S2E), 4.8 ± 0.2 $Å^2/(e^-/Å^2)$ (Fig. S7A), and 4.9 ± 0.3 $Å^2/(e^-/Å^2)$ (Fig. S7A). We note that the first few frames were excluded from these fits since their B-factors did not decay linearly, even though we measured no movement in gold nanoparticles. The cause of this was found to be intermittent charging artefacts that were present to varying degrees on all datasets collected on this microscope, irrespective of the specimen support geometry or the specimen temperature. Based on previously published data (4), we do not expect this to be present in data collected on a newer TEM. On measuring the B-factor decay rate at a specimen temperature of 13K, we obtained values of 4.0 ± 0.1 $Å^2/(e^-/Å^2)$, 3.2 ± 0.2 $Å^2/(e^-/Å^2)$, and 3.7 ± 0.5 $Å^2/(e^-/Å^2)$, giving improvements of 1.3×, 1.5×, and 1.3×, respectively. We have plotted the values taken from the first 100 nm hole diameter dataset in Fig. 4.

We also determined the structure of DPS at temperatures of both 81K and 13K to resolutions of 2.4 Å and 2.3 Å, respectively. We performed per-frame reconstructions and fitted a B-factor to the amplitudes between 9–3 Å. Similarly to apoferritin, this resolution range was chosen because to the high resolution achieved in the per-frame maps. We measured a B-factor decay rate of 5.1 ± 0.2 $Å^2/(e^-/Å^2)$ for the 81K dataset and 3.3 ± 0.2 $Å^2/(e^-/Å^2)$ for 13K, giving a 1.5× reduction in the rate of radiation damage for using liquid-helium cooling for this specimen.

The rates of B-factor decay or diffraction pattern spot fading are representative of the average rate of radiation damage to the macromolecules. Certain functional groups of biological macromolecules, such as metal ions, disulfide bonds, and carboxyl groups, damage at a faster rate than the average of the molecule (6). Using X-ray crystallography, it has been suggested that liquid-helium cooling can reduce the rate of damage to disulfide bonds in lysozyme by a factor of 4 (7). Investigating this is beyond the scope of this work, but if it is true in cryo-EM, it will provide an even greater benefit to liquid-helium cooling.

Specimen motion.

Specimen motion at the onset of irradiation causes a blurring of the images and subsequent reduction in data quality. The effect of this is particularly apparent in per-frame B-factor plots (Fig. S2E), where the B-factors do not decay linearly due to uncorrected motion in the initial frames. Although motion can be partially corrected by using techniques like motion correction or Bayesian Polishing (8), these methods are only effective when there is sufficient signal in each frame and no significant motion within the timescale of the frame. Rapid motion caused by the relaxation of the foil (2) and buckling of the frozen water (4) is too fast (in the fluence, not the time domain) to be fully corrected.

At liquid-nitrogen temperatures, beam-induced specimen motion becomes negligible when all-gold grids are used and when the hole diameter to frozen water thickness aspect ratio is <11:1 (4). This is because the movement is caused by expansion of the frozen water that causes it to buckle, and smaller holes or a thicker sample will increase the energetic barrier to buckling and leave the frozen water under stress. For apoferritin samples in 2 μm diameter hole

UltrAuFoil grids, we can estimate the motion and subsequent B-factor based on measurements of gold nanoparticle tracking at 81K (4) and studies showing that motion is approximately twice as severe at liquid-helium temperatures compared to liquid-nitrogen temperatures (9). When reducing the hole size to 300 nm (aspect ratio ≈10:1) and irradiating with a ≈ 1 μm diameter beam, our data in Fig. S2 suggests that the B-factor is approximately 35 Å^2 more negative in the first frame at 13K compared to 81K, with the motion reducing until it becomes negligible at around 5 e$^-$/Å^2. For 100 nm diameter holes (aspect ratio <5:1) and a 300 nm diameter beam, the movement does not significantly contribute to the B-factor. We suspect that the reduction in buckling aspect ratio threshold at liquid-helium temperatures as compared to liquid-nitrogen temperatures is caused by expansion of the frozen water during the first 5 e$^-$/Å^2 of irradiation. Buckling is unlikely to be due to the contraction of the gold foil, which would reduce the hole diameter by less than 1 nm (10). Furthermore, it is unlikely that the frozen water buckles during cooling to liquid-helium temperatures from liquid-nitrogen temperatures, as both our EELS measurements (Fig. 2C) and previous studies (11, 12) suggest little to no change in density.

The driving mechanism behind the expansion of the frozen water remains uncertain, as the final structure of the frozen water is not well understood. It is counterintuitive that the diffraction pattern peak is shifting to a higher resolution (smaller spacing), yet the volume expands. We hypothesize that species with oxygen atoms in closer proximity than in LDA—possibly rearranged water surrounding radiation damaged species or even hydrogen peroxide (Fig. S4), known to increase in concentration during irradiation at liquid-helium temperatures (13)—could be responsible. The broadening and weakening of the water ring in the diffraction patterns suggest that the frozen water is becoming more varied, likely as the concentrations of radiolytic species increase (13) due to reduced rates of recombination caused by their restricted mobility. The reduced escape of hydrogen gas from the sample is a particularly striking example of this (Movies S3 & S4). The combination of previously gaseous species no longer escaping from the sample and a transition from covalent to van der Waals bonding as recombination rates decrease may be driving the observed expansion. Overall, we propose that the structure can no longer be considered as merely water molecules, but a complex mixture of frozen, hydrated, amorphous radiolytic species. Nonetheless, further experiments are needed to fully understand the structure of amorphous water after electron irradiation at liquid-helium temperatures and to reconcile the apparent contradiction between the diffraction, EELS, and nanoparticle tracking data. Namely, the water expands in the nanoparticle tracking experiments, but this expansion was not detected in the EELS experiments. This could be because the expansion was too small to detect given the accuracy of the peak shift measurements but it could also be related to the complex and unknown structure of water after irradiation when the fragments generated are trapped.

Charge accumulation.

Specimen charging has long been recognized as a significant issue in electron microscopy of insulating materials (14–17), including amorphous frozen water. When a high-energy electron beam irradiates an insulating material, inelastic scattering generates secondary electrons that may escape into the vacuum, leaving behind a positive charge (18). Since the material is insulating, these electron holes are not immediately filled, leading to positive charge accumulation. A charge equilibrium is eventually reached due to the contribution of neutralizing secondary electrons from the surroundings, reduced escape of secondary electrons caused by positive charge accumulation, and possibly electrical breakdown of the insulator to render it conductive (18, 19). This positive charge causes the specimen to act as a lens, altering the phase of the incoming electron wavefront—a phenomenom referred to as the 'Berriman effect' (20)—potentially leading to micrograph blurring.

When irradiating amorphous frozen water alone (i.e. no foil) at ≈80K, charge accumulation occurs over the first ≈ 1.5 e$^-$/Å^2 of irradiation (19), causing fields on the order of 10^8 V/m (21). However, irradiating a conductive material as well as the frozen water significantly reduces the magnitude of positive charge accumulation (15, 19, 22–25). Our first goal was to ensure that the support foil remains conductive at liquid-helium temperatures. We measured the resistivity of the gold foil, amorphous carbon foil, and carbon rod at room temperature, 77K, and 4K using a cryo-four-point probe instrument (Fig. S9A). The results, shown in table S1, confirm that at liquid-helium temperatures, the gold foil is more conductive than at liquid-nitrogen temperatures, making it unlikely to accumulate charge. In contrast, the conductivity of the carbon rod and amorphous carbon foil decreases with decreasing temperature. Notably, we were unable to measure any resistance for the amorphous carbon foil at 4K, indicating that it becomes insulating and should not be used for cryo-EM at liquid-helium temperatures.

Having established that the gold foil is conductive at liquid-helium temperatures, our next objective was to determine if charge accumulation in the frozen water still saturates before a fluence of 0.1 e$^-$/Å^2. We measured the contraction of a convergent beam at the onset of irradiation (Fig. S10). Although the beam size reduced more at the lower specimen temperatures, suggesting positive charge accumulation was greater, it still saturated well before 0.1 e$^-$/Å^2. This suggests that, similar to observations at liquid-nitrogen temperatures, charge accumulation is unlikely

to significantly contribute to the B-factor.

Microscopic charge fluctuations.

The characteristic 'bee-swarm' effect seen in highly defocused micrographs of insulating specimens (14), such as amorphous frozen water, is caused by microscopic charge fluctuations (15). This becomes convoluted with the spatial coherence envelope function to add a defocus-dependent envelope function to the micrographs. The effect of this was determined by Russo and Henderson (26) by measuring the defocus-dependent fading of the power in the 111 ring of gold nanoparticles in frozen water compared to a dry gold foil. We have repeated the same measurement at liquid-helium temperatures and fitted the fading to a spatial coherence envelope function (Fig. S12). The difference in apparent semi-angle in the envelope function (equation 4) between the dry foil and foil/gold particle is as a result of charge fluctuations. The magnitude of this difference is very similar at both 81K and 13K. The gold nanoparticle dataset did not have the same apparent semi-angle as the foil+frozen water dataset, which is likely due to the rotation of the gold nanoparticles during irradiation. Nonetheless, the effect of charge fluctuations is clearly small in all cases. At a standard defocus for single-particle data collection (< 2 μm), the microscopic charge fluctuation envelope function contributes a B-factor of < 1 Å^2 for both specimen temperatures.

Pseudo-Brownian motion.

Energy deposited by the primary electron beam into the amorphous frozen water induces the movement of water molecules. The molecular motion subsequently causes displacement of the embedded protein molecules, with smaller proteins expected to move more significantly than larger ones. This random motion, which continues throughout the exposure, contributes an extra B-factor to the imaging process. Under irradiation by a 300 keV electron beam at liquid-nitrogen temperatures, McMullan *et al.* measured the mean squared displacement (MSD) of water molecules to be ≈ 1.1 Å^2/(e$^-$/Å^2). It has been proposed that frozen water may flow more readily under irradiation at liquid-helium temperatures compared to liquid-nitrogen temperatures (27, 28), potentially resulting in a higher MSD. To investigate this, we replicated the methodology of McMullan *et al.* to measure pseudo-Brownian motion at specimen temperatures of both 13K and 81K. We successfully obtained Thon ring patterns at both temperatures (Fig. S11), indicating that the water molecules are moving a similar amount at both temperatures. When quantifying this, we obtained an MSD of 0.87 ± 0.03 Å^2/(e$^-$/Å^2) at 81K and 0.90 ± 0.03 Å^2/(e$^-$/Å^2) at 13K.

The B-factor contributed by pseudo-Brownian motion is therefore similar at both temperatures. To convert the MSD of water molecules to a B-factor, we first estimate the MSD of a protein molecule. The MSD relates to the diffusion coefficient, D, as follows

$$\mathrm{MSD} = 2nDt \qquad \text{[S1]}$$

where t is time and n the number of dimensions. The diffusion coefficient is related to the diameter of a spherical particle by the Stokes-Einstein equation (29):

$$D = \frac{k_B T}{6\pi\eta r} \qquad \text{[S2]}$$

where k_B is the Boltzmann constant, T the absolute temperature, η the dynamic viscosity, and r the particle radius. Assuming the protein is spherical, the radius can be estimated as

$$r = \left(\frac{3M_w}{4\pi\rho}\right)^{\frac{1}{3}} \qquad \text{[S3]}$$

where M_w is the molecular mass and ρ is the protein density, estimated as 0.8 Da/Å^3. The MSD of a protein molecule can be converted to a Debye-Waller factor (30, 31), which is equivalent to a B-factor, using equation S4.

$$B = \frac{8\pi^2}{3}\mathrm{MSD} \qquad \text{[S4]}$$

Using a water molecule diameter of 2.7 Å, we can estimate the B-factor for a range of different protein sizes (Table S2). For a 100 kDa protein over an exposure of 25 e$^-$/Å^2, this would add a B-factor of 25 Å^2, which is plotted in Fig. 4. We note that this analysis assumes that the protein molecules remain intact during irradiation. In reality, radiolysis causes both fragmentation and cross-linking, and these events complicate any discussion of pseudo-Brownian motion.

Bubbling.

Hydrogen gas bubbles are known to be produced by electron irradiation of protein samples in single-particle cryo-EM (33). However, this typically occurs only after fluences of several hundreds of e$^-$/Å^2, meaning they are of little concern. At liquid-helium temperatures, hydrogen gas bubbling becomes more pronounced (Movies S3 & S4), and

previous studies have reported that this can reduce contrast in micrographs (34). In our datasets, although hydrogen gas bubbling is more vigorous at liquid-helium temperatures compared to liquid-nitrogen temperatures, it still only becomes apparent after several hundreds of $e^-/Å^2$ (Movies S3 & S4). Therefore, it does not contribute any additional B-factor to imaging and explains why we saw no change in particle-image contrast when imaging at liquid-helium temperatures. It is important to note that the onset of bubbling may vary depending on the specific molecules or buffers present and their concentrations (25). When using liquid-helium cooling for cellular imaging, further investigation will be required to determine whether hydrogen gas bubbling poses an issue.

Practicalities and throughput.

It is perhaps obvious that using 100 nm diameter holes reduces the throughput of data collection compared to larger holes where more particles can be collected per micrograph. We also found in the case of apoferritin, but not DPS, that preparing specimens with thin ice can be more challenging. Our apoferritin had a propensity to aggregate on the edge of the specimen support foil, which was mitigated, but not eliminated, by treatment of the foil with $FeCl_3$ and potassium citrate. To increase throughput, it may be that the aspect ratio limit at helium temperatures can be increased by using a continuous specimen support, such as graphene (35). We may also be able to increase the throughput by packing the 100 nm holes closer together to enable multiple holes to be imaged in a single micrograph on a large area detector. This will require reducing the stress present in the foil, potentially by fabricating grids entirely from metals that contract less at low-temperatures, such as molybdenum or titanium, or by relieving foil stress through rapid heating and revitrification of the frozen water at liquid-helium temperatures using a laser (36). Regardless, the high degree of automation available in modern cryomicroscopes means that even using very small holes, as described above, would be practical for most specimens.

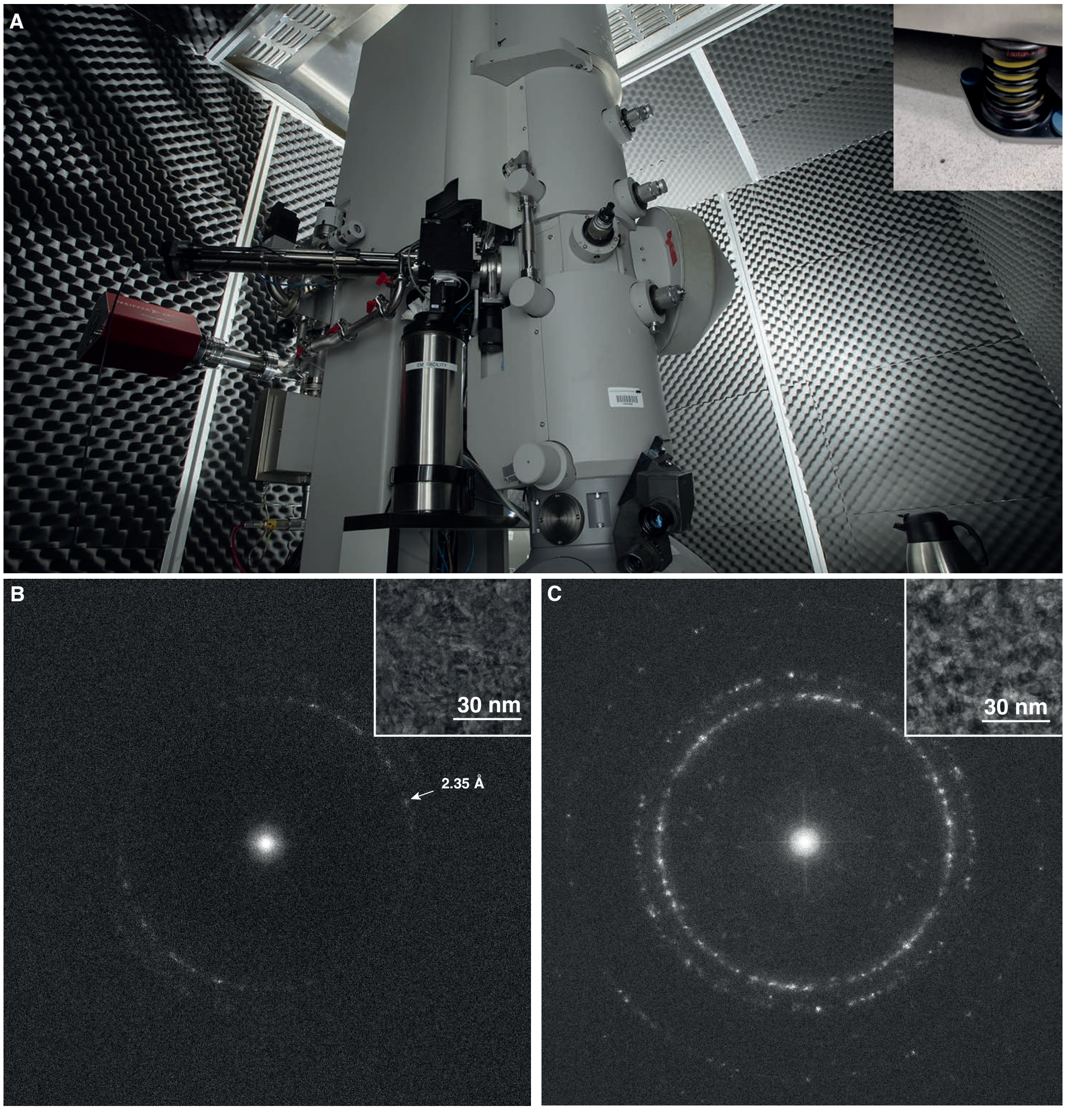

Fig. S1. The Polara G2 TEM that was used for data collection at liquid-helium temperatures. (**A**) The TEM is inside an aluminium enclosure to reduce the impact of airflow on the microscope. The enclosure is lined with acoustic foam to dampen acoustic noise. The inset shows spring vibration isolation feet that the enclosure sits on to isolate it from vibrations in the ground and, along with louvred ceiling panels, allow vapour to circulate passively through the enclosure. The photo was provided by Neil Grant, MRC-LMB. Micrographs of a polycrystalline gold foil (insets) were taken at a fluence of ≈ 80 e$^-$/Å^2, and corresponding Fourier transforms before (**B**) and after (**C**) the installation of the enclosure are shown.

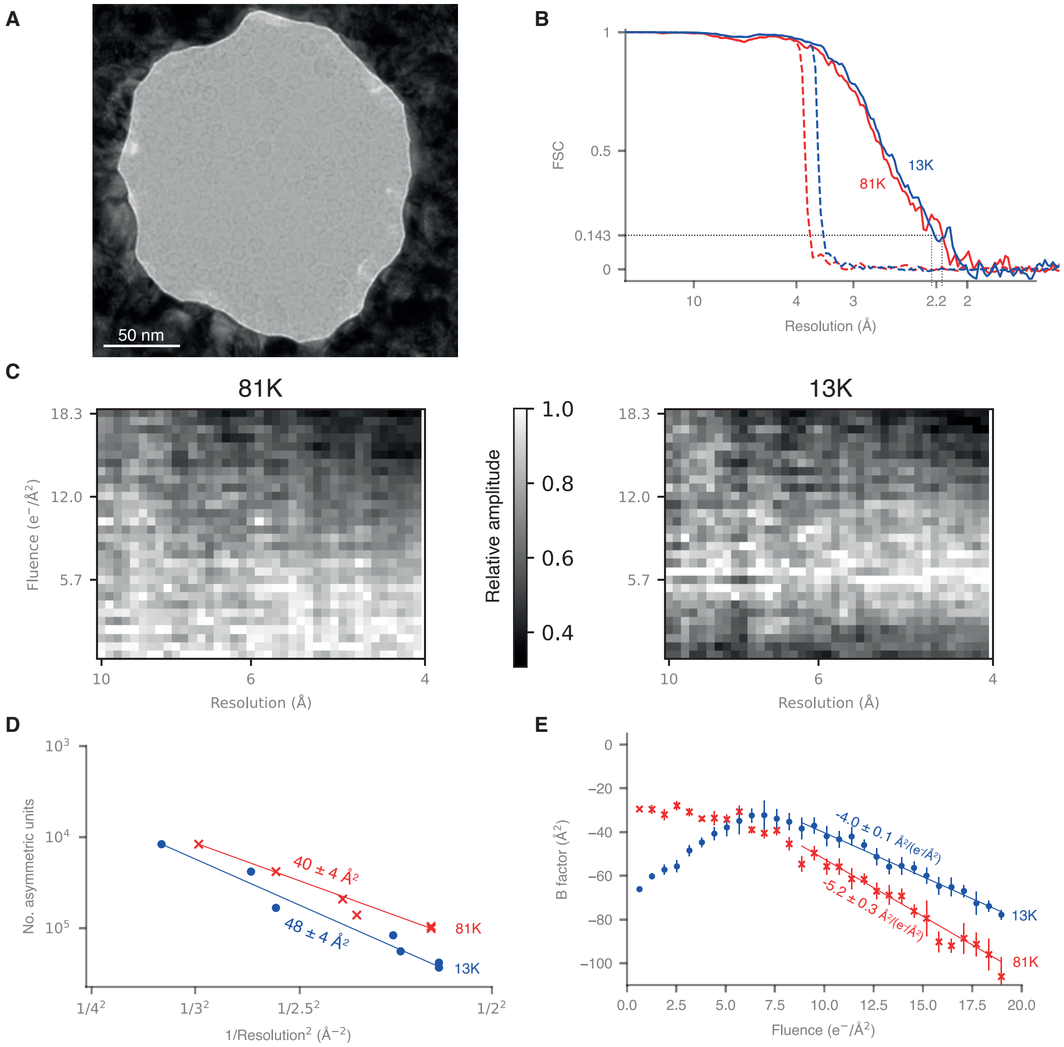

Fig. S2. Apoferritin structures on 200 nm hole diameter HexAuFoil grids. (**A**) An example micrograph of apoferritin in a 200 nm diameter hole. (**B**) The FSC curves (masked maps) for data collected at 13K (blue) and 81K (red). The dashed lines are the phase randomized FSCs. (**C**) The plots of the amplitude ratio between the per-frame maps and the first frame (equation 1). Each pixel represents the amplitude ratio between the frame at a particular fluence to the highest amplitude frame for a given resolution shell. (**D**) Rosenthal *B*-factor plots for the two datasets (number of asymmetric units vs frequency squared) are shown with the corresponding *B*-factors estimated from the straight line fits. (**E**) The slopes of the linear fits to the natural logarithm of the amplitude ratios (equation 1, resolution range 9–3 Å)) with the intercepts restrained to be 0 for each dataset. The error bars are the standard error of the mean.

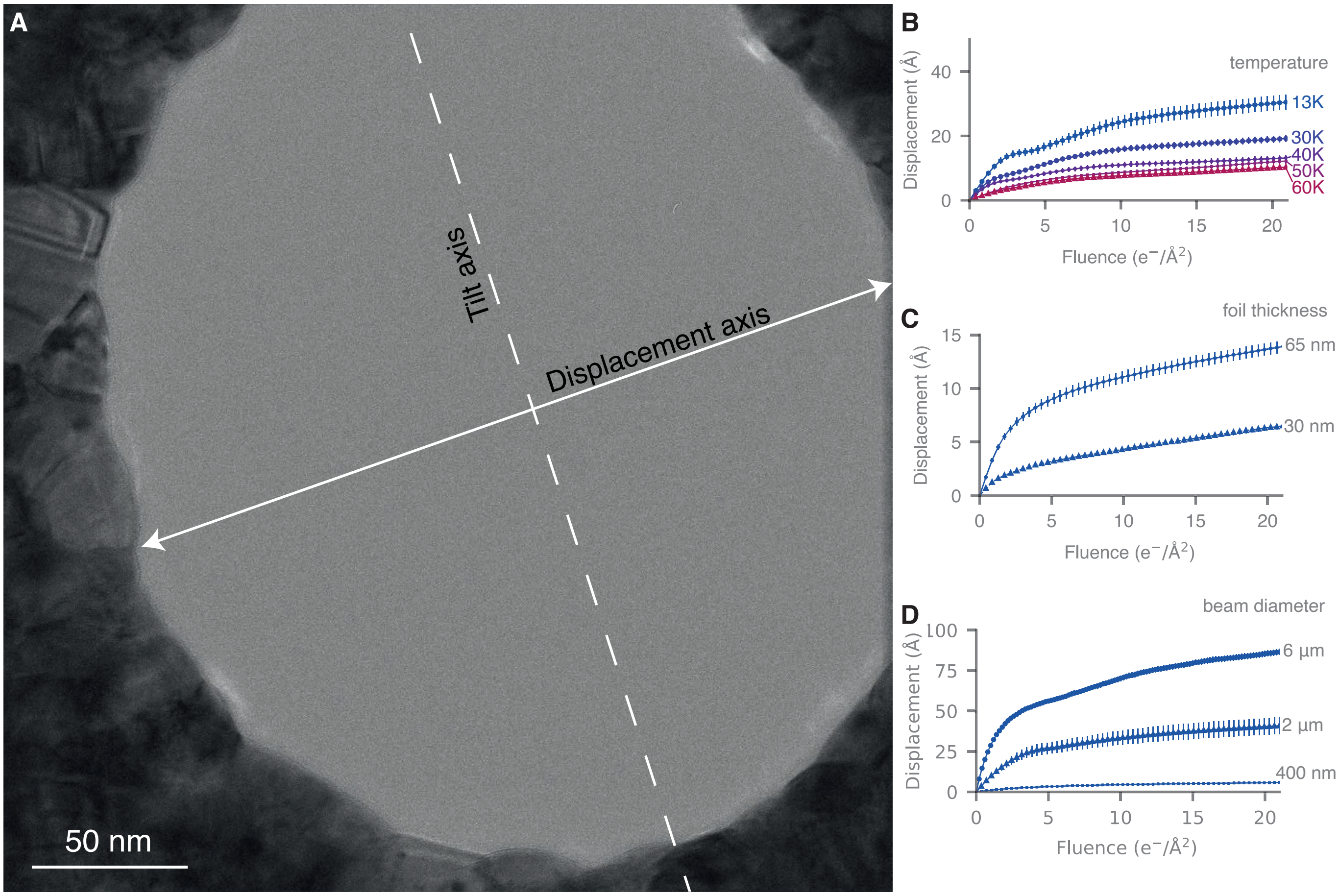

Fig. S3. Experiments to characterize the movement of gold foils. (**A**) The movement of the gold foil was measured as the mean cumulative motion, as determined by motion correction, along the axis perpendicular to the tilt axis, (labeled as 'displacement axis'). The specimens were apoferritin on 300 nm hole diameter gold grids and the specimen temperatures were 13K, beam sizes 1.2 μm, and foil thicknesses 30 nm, unless otherwise specified. Measurements were made for different (**B**) specimen temperatures, (**C**) foil thicknesses, and (**D**) beam diameters. All errors are the standard error of the mean.

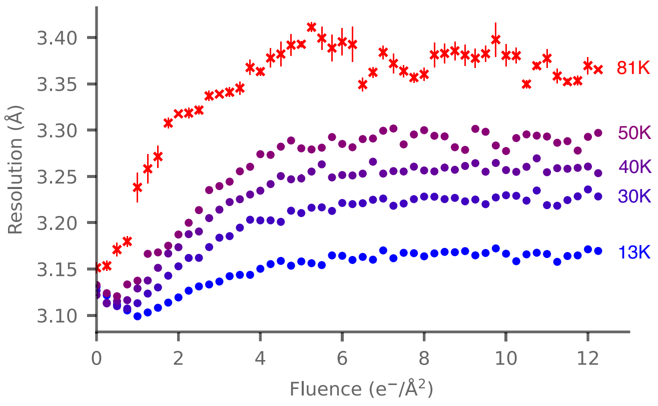

Fig. S4. Electron diffraction of 30% hydrogen peroxide. The plot shows the diffraction peak position of 30% hydrogen peroxide as a function of fluence for different specimen temperatures. The error bars are the standard error of the mean.

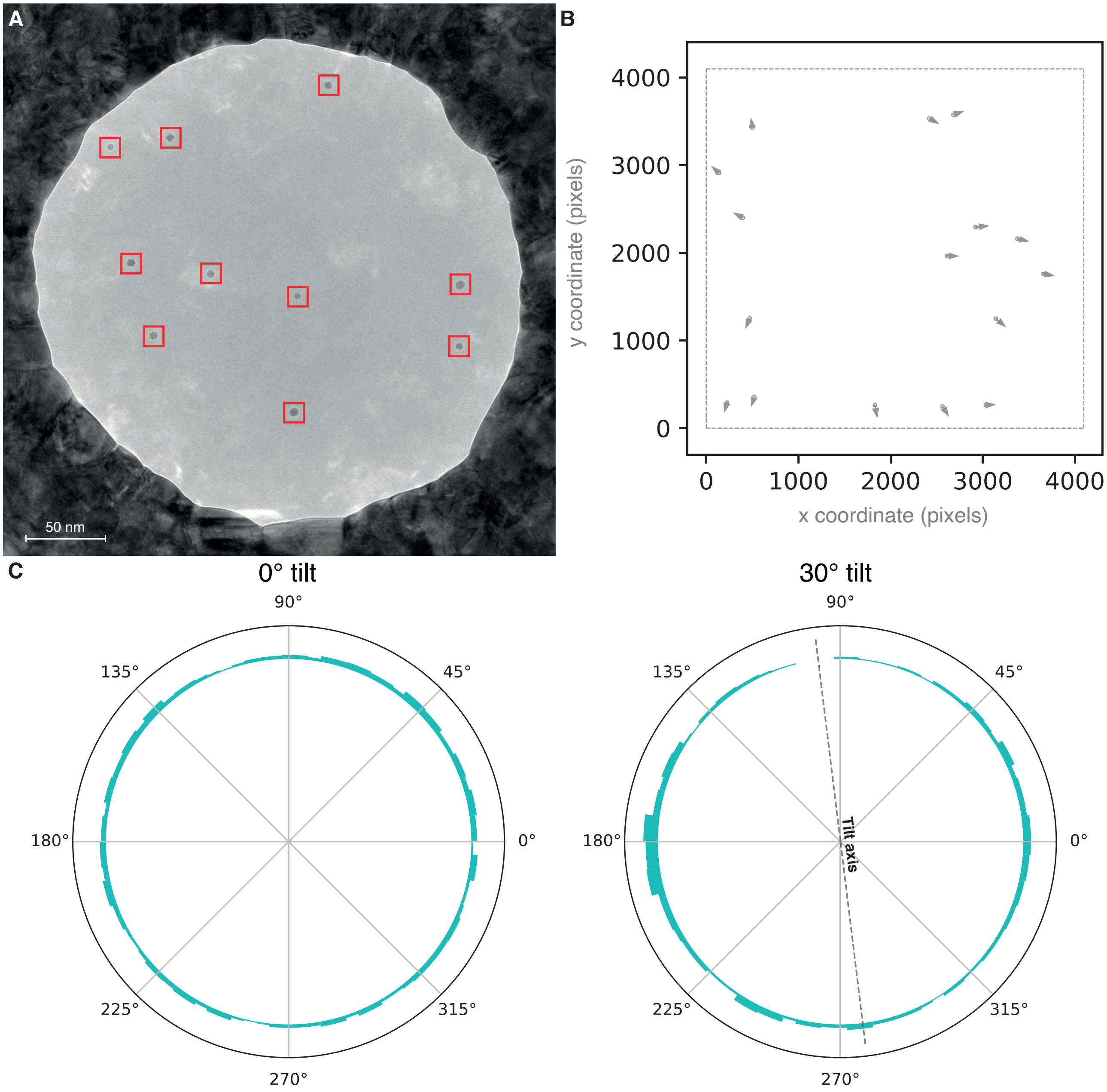

Fig. S5. Movement tracking of 5 nm gold nanoparticles on 300 nm hole diameter HexAuFoil grids. (**A**) An example micrograph is shown. (**B**) Particle trajectories of nanoparticles in a single hole show that the particles move away from the centre. (**C**) The histograms show the trajectories for particles with the stage un-tilted and tilted to 30°. In the tilted dataset, the trajectories are biased perpendicular to the tilt axis, suggesting a motion along the beam axis consistent with buckling.

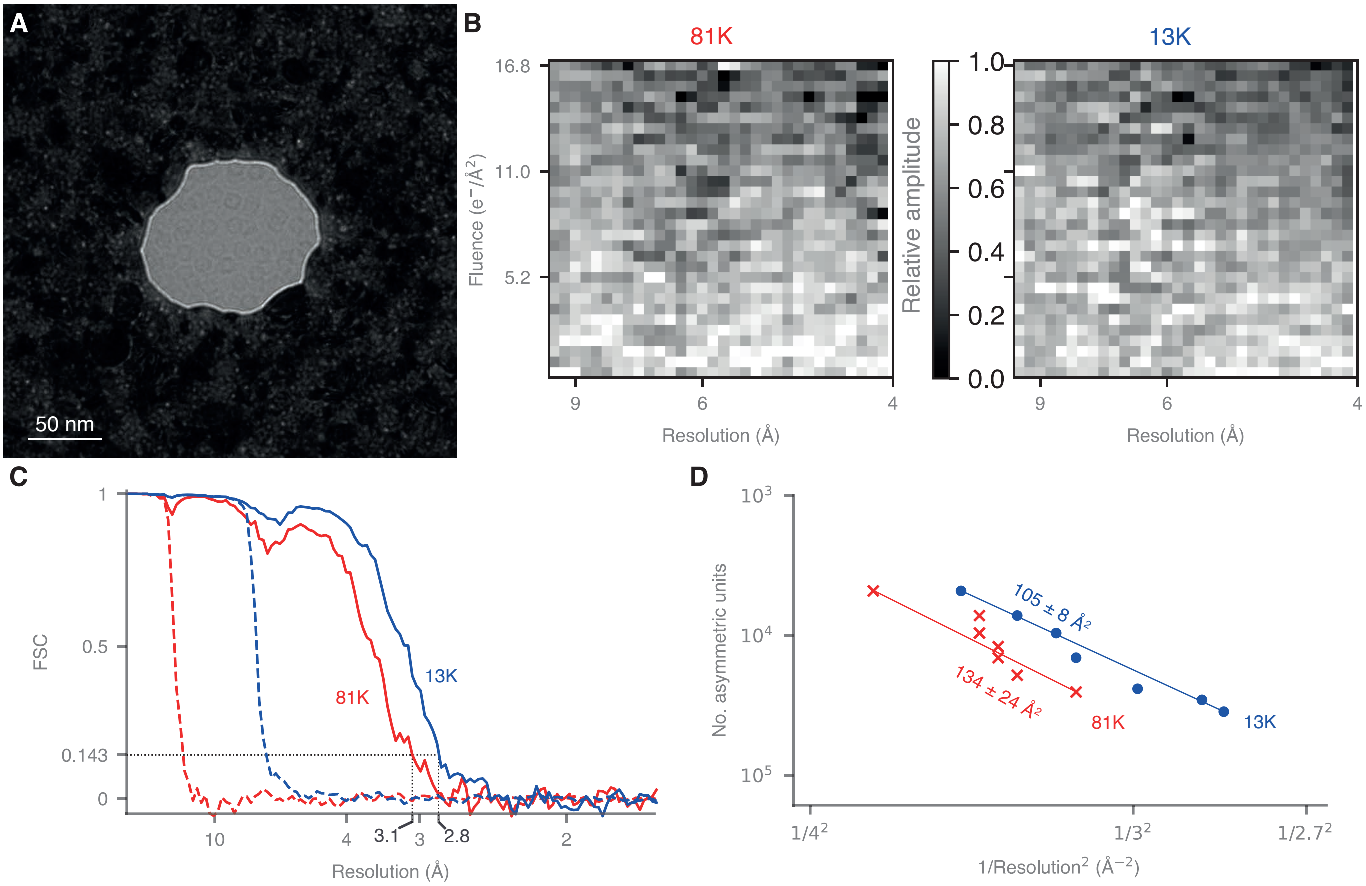

Fig. S6. Apoferritin structures on 100 nm hole diameter HexAuFoil grids. (**A**) An example micrograph of apoferritin in a 100 nm diameter hole is shown. (**B**) The plots show the amplitude ratio between the per-frame maps and the first frame (equation 1). Each pixel represents the amplitude ratio between the frame at a particular fluence to the highest amplitude frame for a given resolution shell. (**C**) The FSC curves for data collected at 13K (blue) and 81K (red) are shown. The dashed-lines are the phase randomized FSCs. (**D**) Rosenthal *B*-factor plots for the two datasets (number of asymmetric units vs frequency squared) and the corresponding *B*-factors estimated from the straight line fits are shown. Note that for the same number of particles, the resolution of the structures increased at 13K from 3.1 to 2.8 Å.

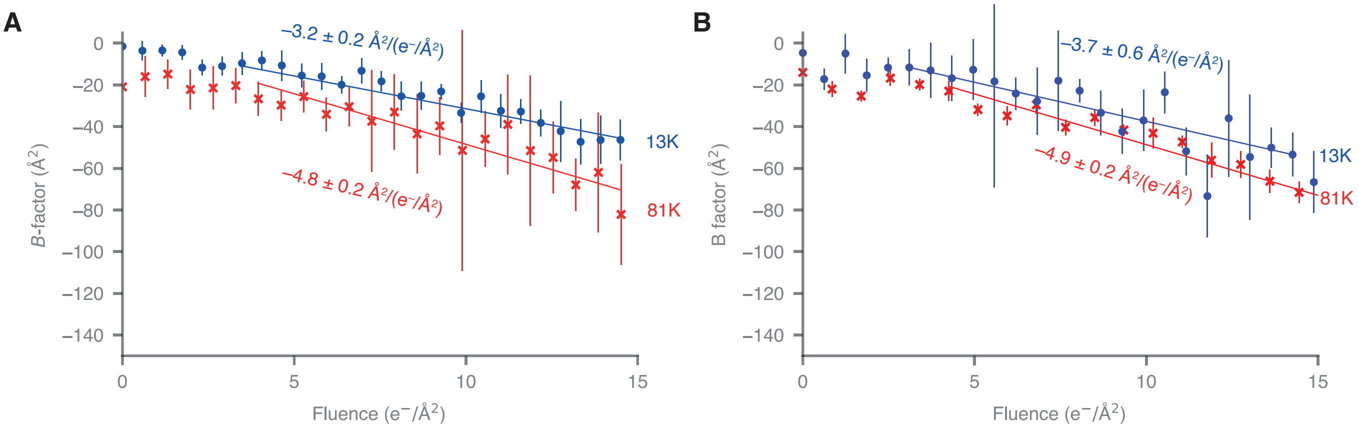

Fig. S7. Per-frame *B*-factors for apoferritin structures on 100 nm hole diameter HexAuFoil grids. The resolution range for fitting the decay was 10–4 Å. The error bars are the standard deviation. The liquid-helium cooled datasets damaged slower than the liquid-nitrogen datasets by factors of $1.5 \pm 0.1 \times$ (**A**) and $1.3 \pm 0.2 \times$ (**B**).

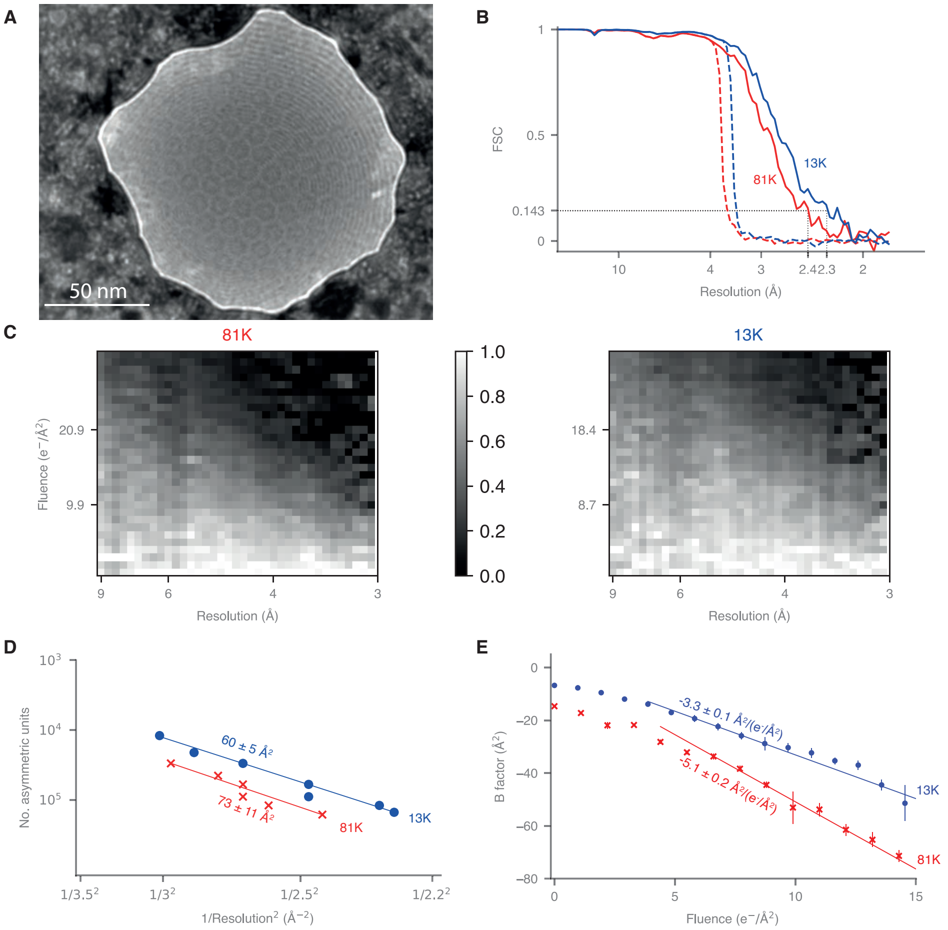

Fig. S8. DPS structures on 100 nm hole diameter HexAuFoil grids. (**A**) An example micrograph of DPS in a 100 nm diameter hole is shown. (**B**) The plot shows FSC curves for data collected at 13K (blue) and 81K (red). The dashed lines are the phase randomized FSCs. (**C**) The amplitude ratio between the per-frame maps and the first frame (equation 1) is shown. Each pixel represents the amplitude ratio between the frame at a particular fluence to the highest amplitude frame for a given resolution shell. (**D**) Rosenthal *B*-factor plots for the two datasets (number of asymmetric units vs frequency squared) are shown with the corresponding *B*-factors estimated from linear fits. (**E**) The plot shows the slopes of the linear fits to the natural logarithm of the amplitude ratios (equation 1, resolution range 9–3 Å) with the intercepts restrained to be 0 for each dataset. The error bars are the standard error of the mean. Note that for the same number of particles, the resolution of the structures increased at 13K from 2.4 to 2.3 Å.

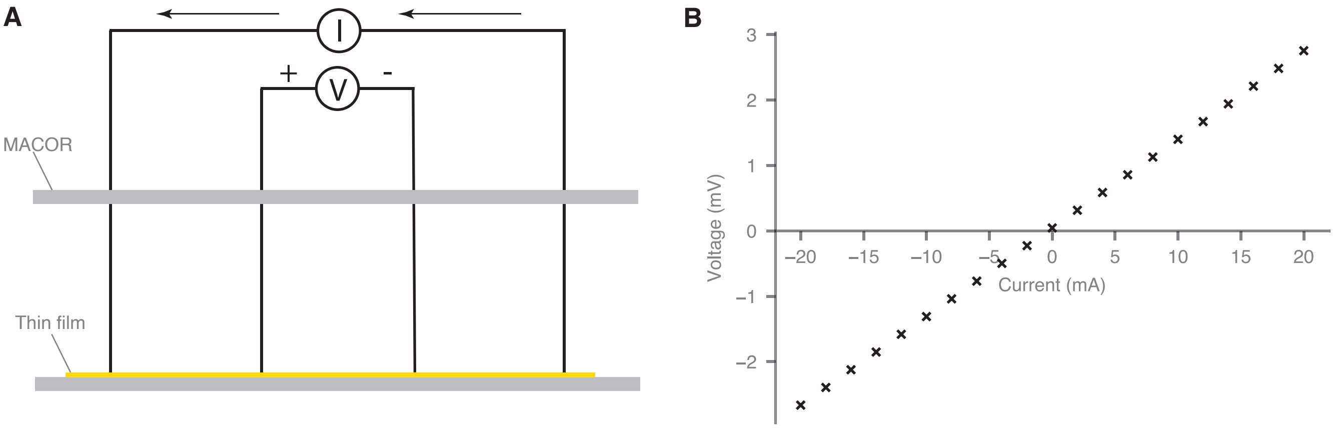

Fig. S9. Measuring the resistivity of polycrystalline gold foils used in cryo-EM specimen supports. (**A**) The schematic diagram depicts the cryo-four-point probe instrument used for the resistivity measurements. The resistance was measured for a 30 nm thick gold foil, 50 nm thick amorphous carbon, and a 3 mm diameter carbon rod. In the experiment, current was sourced, and voltage was measured. An example output for the gold foil at 4K is plotted in (**B**).

Sample	Temperature (K)	Resistance (Ω)	$\sigma_{\bar{x}}$ (Ω)
Gold foil	295	3.60×10^{-8}	3.11×10^{-12}
Gold foil	77	1.52×10^{-8}	7.98×10^{-13}
Gold foil	4	9.56×10^{-9}	1.32×10^{-13}
Carbon rod	295	1.28×10^{-5}	7.34×10^{-10}
Carbon rod	77	2.07×10^{-5}	1.46×10^{-8}
Carbon rod	4	2.49×10^{-5}	2.94×10^{-10}
Amorphous carbon foil	295	0.146	0.0065
Amorphous carbon foil	77	8.50	0.033
Amorphous carbon foil	4	-	-

Table S1. Resistance measurements for three different specimens at room temperature, liquid-nitrogen temperature, and liquid-helium temperature. The gold foil was 30 nm thick, the amorphous carbon 50 nm thick, and the carbon rod a diameter of 3 mm. $\sigma_{\bar{x}}$ is the standard error of the mean. The gold foil becomes more conductive as the temperature reduces, whereas the carbon becomes less conductive. We were unable to obtain a reliable measurement for the resistance of the amorphous carbon film at 4K.

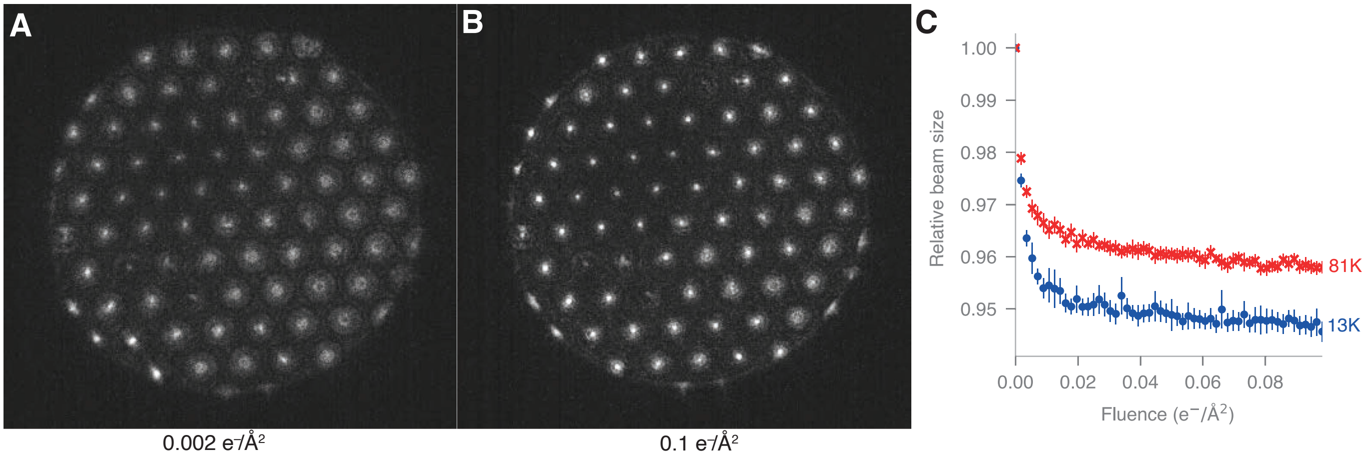

Fig. S10. Measuring charge accumulation during cryo-EM imaging. Low-magnification $(2150\times)$ images of a section of a grid-hexagon from a R0.3/0.3 HexAuFoil grid at 5 mm underfocus and after a fluence of (**A**) 0.002 $e^-/Å^2$ and (**B**) 0.1 $e^-/Å^2$ are shown. Each hole contains a specimen of apoferritin in amorphous frozen water. The low-magnification ensures the entire beam can be seen and that very high-defocus can be achieved with the first intermediate lens. The positive charge accumulation causes a change in beam size as well as a change in defocus and magnification. (**C**) The plot shows the change in beam size relative to the first frame, with the error bars being the standard error of the mean. The beam size reduces more in the 13K dataset but still begins to plateau within the first 0.1 $e^-/Å^2$.

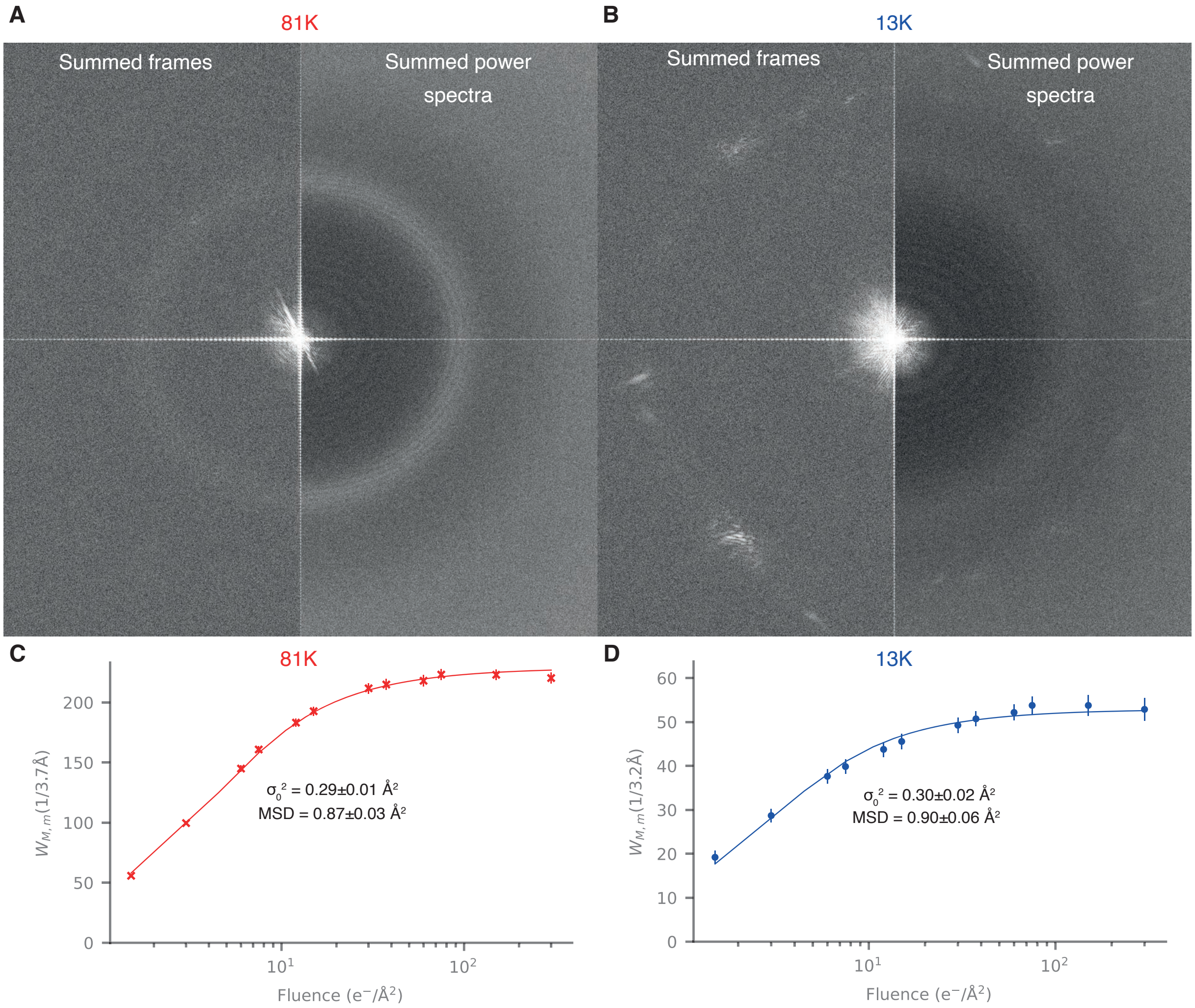

Fig. S11. Pseudo-Brownian motion of water molecules at a beam energy of 300 keV. The pseudo-Brownian motion of water molecules was measured according to the method of McMullan *et al.* (37). Power spectra of amorphous frozen water collected at specimen temperatures of 81K (**A**) and 13K (**B**) are shown. The left side of each is the power spectrum of the sum of the frames and the right side is the sum of the power spectra of each individual frame. The Thon rings are clearly visible at both temperatures for the summed power spectra. The fits to the peak intensity in the water ring are shown in (**C and D**), which are used to estimate the mean squared displacement (MSD) of the water molecules. The error bars are the standard error of the mean.

M_w (kDa)	r Å	MSD Å^2/(e$^-$/Å^2)	*B* Å^2/(e$^-$/Å^2)
10	14.4	0.084	2.2
50	24.6	0.049	1.3
100	31.0	0.039	1.0
250	42.1	0.029	0.76
500	53.0	0.023	0.60
1000	66.8	0.018	0.48
2000	84.2	0.014	0.38

Table S2. The predicted mean squared displacement (MSD) and Debye-Waller *B*-factor for globular proteins of different molecular masses (M_w) and radii (r) are tabulated. This is based on a water molecule diameter of 2.7 Å and a water molecule MSD of 0.9 Å^2/(e$^-$/Å^2).

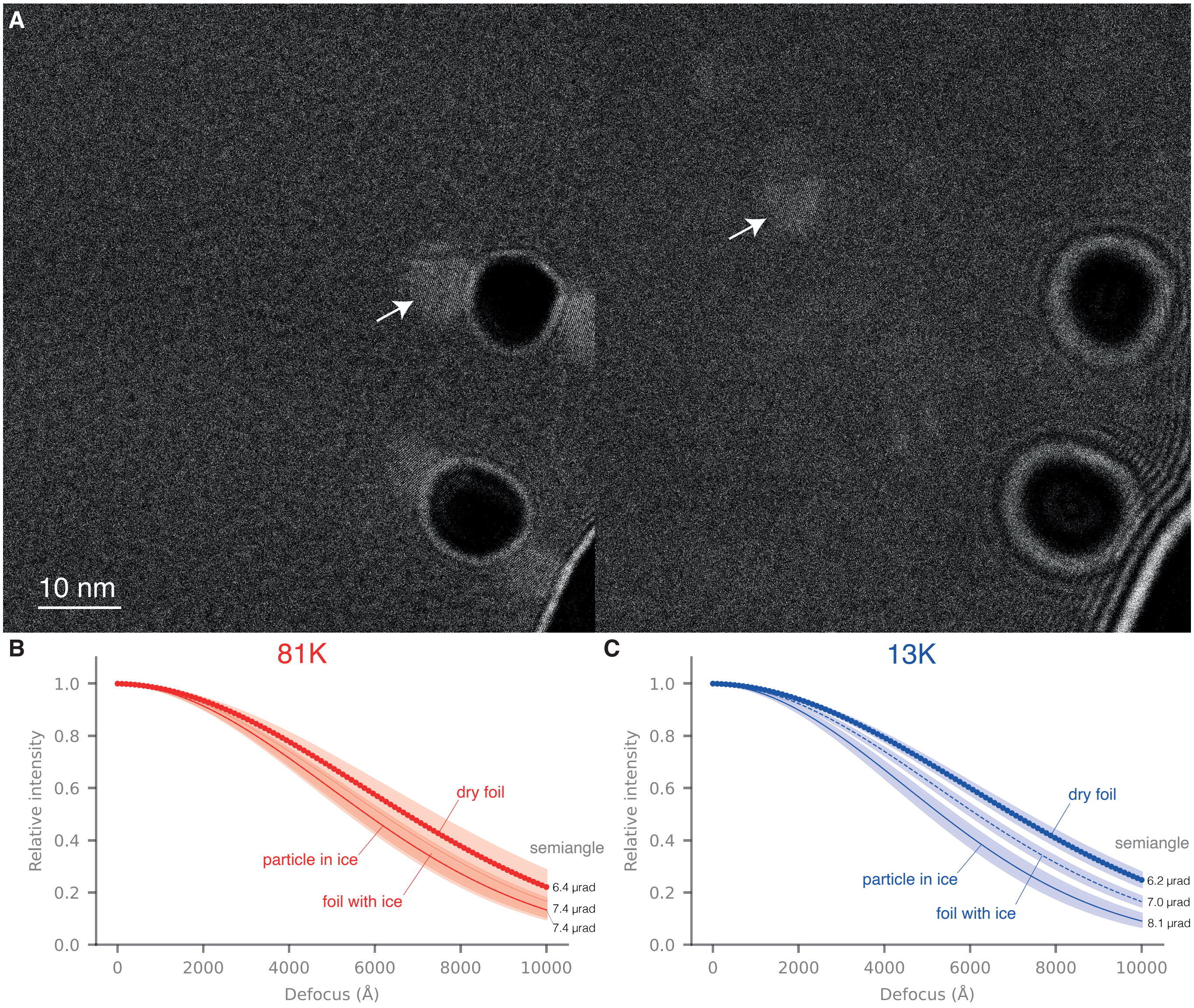

Fig. S12. Measurements of microscopic charge fluctuations. (**A**) Specimens of gold nanoparticles in frozen water on all-gold grids were imaged at low-defocus (left) and high-defocus (right). The fading of the gold 111 ring as a function of defocus was measured for gold nanoparticles, the foil in frozen water, and a dry foil. This was fitted to a spatial coherence envelope function and plotted for specimen temperatures of 81K (**B**) and 13K (**C**). The small difference in envelope function with and without frozen water indicates that charge fluctuations have a negligible impact on image quality at both temperatures.

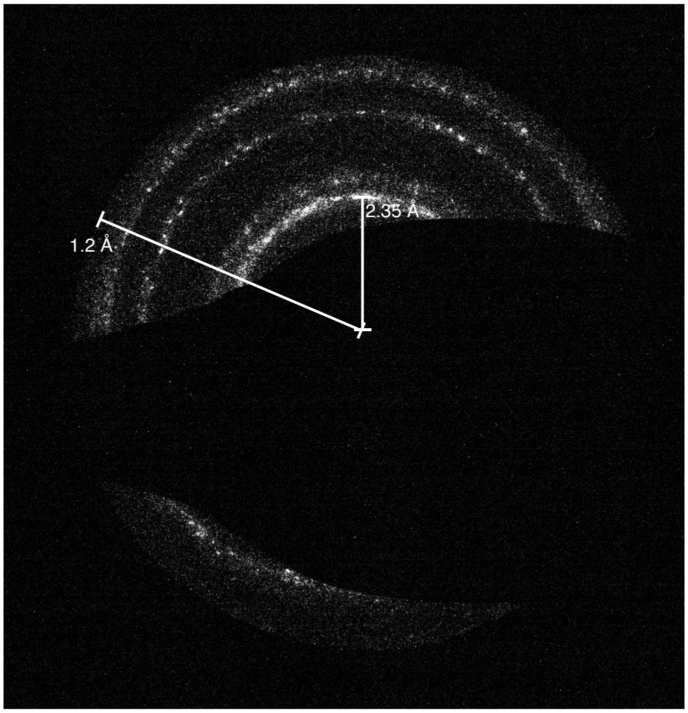

Fig. S13. Diffraction pattern of the gold foil of a HexAuFoil R0.3/0.3 with a 100 μm diameter objective aperture inserted. This was used to calibrate the size of the objective aperture in reciprocal space.

Specimen	Hole Diameter (nm)	Temp. (K)	Exp. No.	Pixel size (Å)	Fluence/Frame ($e^-/Å^2$)	Total Fluence ($e^-/Å^2$)	Exposure Time (s)	No. Movies	Defocus Range (µm)	Avg. Defocus (µm)	No. Particles	Symmetry	Resolution FSC 0.143 (Å)	Damage Rate ($Å^2/e^-/Å^2$)
ApoF	200	81	1	0.648	0.63	63.5	5	210	-0.6 – -2.2	-1.4	4207	O	2.2	5.2
		13	1	0.648	0.63	63.5	5	228	-0.6 – -1.8	-1.2	11519	O	2.2	4
	100	81	1	0.415	0.66	66	5	182	-1.0 – -2.5	-1.8	1054	O	3.1	4.8
		13	1	0.415	0.58	58	5	206	-1.0 – -1.7	-1.7	1457	O	2.8	3.2
	100	81	2	0.415	0.85	51	3	142	-1.0 – -3.0	-2.2	7002	O	3.0	4.9
		13	2	0.415	0.62	49.6	4	219	-1.0 – -3.0	-2.2	7861	O	3.3	3.7
DPS	100	81	1	0.415	1.1	55	5	228	-0.7 – -1.9	-1.3	13544	T	2.4	5.1
		13	1	0.415	0.97	58.2	6	265	-0.7 – -1.9	-1.3	12545	T	2.3	3.3

Table S3. Summary of data collection and processing parameters for single particle cryo-EM structures determined in this manuscript.

Caption for Movie S1. **59,000× magnification image of the gold foil movement at 13K.** The grid is a 300 nm hole diameter HexAuFoil grid with apoferritin. The sample was irradiated with 300 keV electrons at a flux of 0.4 $e^-/Å^2$/frame. The motion of the image is perpendicular to the tilt axis.

Caption for Movie S2. **31,000× magnification image of the gold foil movement at 13K.** The grid is a 300 nm hole diameter HexAuFoil grid with apoferritin. The sample was irradiated by 300 keV electrons at a flux of 0.4 $e^-/Å^2$/frame. The motion of the image is perpendicular to the tilt axis.

Caption for Movie S3. **Bubbling of apoferritin specimens at temperatures of 81K and 13K** The grid is an R1.2/1.3 UltrAuFoil grid and the specimen apoferritin. The sample is irradiated by 300 keV electrons at a flux of 50 $e^-/Å^2$/frame. Hydrogen bubbling is significantly more abundant at the lower temperature but is still only visible after a fluence of > 100 $e^-/Å^2$.

Caption for Movie S4. **Bubbling of Hepetitis B viral capsid specimens at temperatures of 81K and 13K** The grid is an R1.2/1.3 UltrAuFoil grid and the specimen the capsid from the Hepatitis B virus. The sample is irradiated by 300 keV electrons at a flux of 64 $e^-/Å^2$/frame. Hydrogen bubbling is significantly more abundant at the lower temperature and is still only visible after a fluence of > 100 $e^-/Å^2$ after the high resolution features of the molecules are lost. In addition, the bubbles are clearly associated with the individual capsids at 13K whereas they are only roughly correlated with their positions at 81K. Further work is needed to determine if the hydrogen gas that is generated is from the protein, the water or both.

References

1. Richard Henderson and Christopher J. Russo. Single particle cryoem: Potential for further improvement. *Microscopy and Microanalysis*, 25(S2):4–5, 2019. .
2. Christopher J. Russo and Lori A. Passmore. Ultrastable gold substrates for electron cryomicroscopy. *Science*, 346(6215):1377–1380, 2014.
3. Christopher J. Russo, Steve Scotcher, and Martin Kyte. A precision cryostat design for manual and semi-automated cryo-plunge instruments. *Review of Scientific Instruments*, 87:114302, 2016.
4. Katerina Naydenova, Peipei Jia, and Christopher J. Russo. Cryo-EM with sub–1 Å specimen movement. *Science*, 370:223–226, 2020.
5. Sjors HW Scheres. Beam-induced motion correction for sub-megadalton cryo-EM particles. *eLife*, 3:e03665, aug 2014. .
6. Martin Weik, Raimond B. G. Ravelli, Gitay Kryger, Sean McSweeney, Maria L. Raves, Michal Harel, Piet Gros, Israel Silman, Jan Kroon, and Joel L. Sussman. Specific chemical and structural damage to proteins produced by synchrotron radiation. *Proceedings of the National Academy of Sciences*, 97(2):623–628, 2000. .
7. Alke Meents, Sascha Gutmann, Armin Wagner, and Clemens Schulze-Briese. Origin and temperature dependence of radiation damage in biological samples at cryogenic temperatures. *Proceedings of the National Academy of Sciences*, 107(3):1094–1099, 2010.
8. Jasenko Zivanov, Takanori Nakane, and Sjors H. W. Scheres. A Bayesian approach to beam-induced motion correction in cryo-EM single-particle analysis. *IUCrJ*, 6:5–17, 2019.
9. Olivia Pfeil-Gardiner, Deryck J. Mills, Janet Vonck, and Werner Kuehlbrandt. A comparative study of single-particle cryo-EM with liquid-nitrogen and liquid-helium cooling. *IUCrJ*, 6(6):1099–1105, 2019.
10. Robert J Corruccini and John J Gniewek. Thermal expansion of technical solids at low temperatures; a compilation from the literature:, 1961-01-01 05:01:00 1961.
11. K. Röttger, A. Endriss, J. Ihringer, S. Doyle, and W. F. Kuhs. Lattice constants and thermal expansion of H_2O and D_2O ice I*h* between 10 and 265 K. *Acta Crystallographica Section B*, 50(6):644–648, Dec 1994. .
12. Francesco Mallamace, Caterina Branca, Matteo Broccio, Carmelo Corsaro, Chung-Yuan Mou, and Sow-Hsin Chen. The anomalous behavior of the density of water in the range 30 k < <i>t</i> < 373 k. *Proceedings of the National Academy of Sciences*, 104(47):18387–18391, 2007. .
13. C. Laffon, S. Lacombe, F. Bournel, and Ph. Parent. Radiation effects in water ice: A near-edge x-ray absorption fine structure study. *The Journal of Chemical Physics*, 125(20):204714, 11 2006.
14. D. B. Dove. Image Contrasts in Thin Carbon Films Observed by Shadow Electron Microscopy. *Journal of Applied Physics*, 35(5):1652–1653, 07 1964.
15. G H Curtis and R P Ferrier. The electric charging of electron-microscope specimens. *Journal of Physics D: Applied Physics*, 2(7):1035, jul 1969.
16. J. P. Vigouroux, J. P. Duraud, A. Le Moel, C. Le Gressus, and D. L. Griscom. Electron trapping in amorphous SiO2 studied by charge buildup under electron bombardment. *Journal of Applied Physics*, 57(12):5139–5144, 06 1985.
17. Richard Henderson. Image contrast in high-resolution electron microscopy of biological macromolecules: TMV in ice. *Ultramicroscopy*, 46(1):1–18, 1992.
18. J. Cazaux. Correlations between ionization radiation damage and charging effects in transmission electron microscopy. *Ultramicroscopy*, 60(3):411–425, 1995.
19. Makoto Tokoro Schreiber, Alan Maigné, Marco Beleggia, Satoshi Shibata, and Matthias Wolf. Temporal dynamics of charge buildup in cryo-electron microscopy. *Journal of Structural Biology: X*, 7:100081, 2023.
20. Jacob Brink, Michael B Sherman, John Berriman, and Wah Chiu. Evaluation of charging on macromolecules in electron cryomicroscopy. *Ultramicroscopy*, 72(1):41–52, 1998.
21. John A. Berriman and Peter B. Rosenthal. Paraxial charge compensator for electron cryomicroscopy. *Ultramicroscopy*, 116:106–114, 2012.
22. Robert M. Glaeser and Kenneth H. Downing. Specimen charging on thin films with one conducting layer: Discussion of physical principles. *Microscopy and Microanalysis*, 10(6):790–796, 2004.
23. Brink, Gross, Tittmann, Sherman, and Chiu. Reduction of charging in protein electron cryomicroscopy. *Journal of Microscopy*, 191(1):67–73, 1998.
24. Christopher J. Russo and Richard Henderson. Charge accumulation in electron cryomicroscopy. *Ultramicroscopy*, 187:43–49, 2018.
25. Pilan Zhang, Haiqin Du, Shiwen Cui, Ping Zhou, and Yifei Xu. Response of organic solvents to vitrification and electron exposure in cryo-tem experiments. *Responsive Materials*, 1(2):e20230025, 2023. .
26. Christopher J. Russo and Richard Henderson. Microscopic charge fluctuations cause minimal contrast loss in

cryoEM. *Ultramicroscopy*, 187:56–63, 2018.

27. H.-G. Heide and E. Zeitler. The physical behavior of solid water at low temperatures and the embedding of electron microscopical specimens. *Ultramicroscopy*, 16(2):151–160, 1985.
28. Elizabeth R. Wright, Cristina V. Iancu, William F. Tivol, and Grant J. Jensen. Observations on the behavior of vitreous ice at 82 and 12K. *Journal of Structural Biology*, 153(3):241–252, 2006.
29. Albert Einstein. Über die von der molekularkinetischen theorie der wärme geforderte bewegung von in ruhenden flüssigkeiten suspendierten teilchen. *Annalen der physik*, 4, 1905.
30. P. Debye. Interferenz von röntgenstrahlen und wärmebewegung. *Annalen der Physik*, 348(1):49–92, 1913.
31. Ivar Waller. Zur frage der einwirkung der wärmebewegung auf die interferenz von röntgenstrahlen. *Zeitschrift für Physik*, 17(1):398–408, Dec 1923.
33. Richard D. Leapman and Songquan Sun. Cryo-electron energy loss spectroscopy: observations on vitrified hydrated specimens and radiation damage. *Ultramicroscopy*, 59(1):71–79, 1995.
34. Cristina V. Iancu, Elizabeth R. Wright, J. Bernard Heymann, and Grant J. Jensen. A comparison of liquid nitrogen and liquid helium as cryogens for electron cryotomography. *Journal of Structural Biology*, 153(3): 231–240, 2006.
35. Christopher J. Russo and Lori A. Passmore. Controlling protein adsorption on graphene for cryo-em using low-energy hydrogen plasmas. *Nature Methods*, 11(6):649–652, Jun 2014.
36. Oliver F. Harder, Jonathan M. Voss, Pavel K. Olshin, Marcel Drabbels, and Ulrich J. Lorenz. Microsecond melting and revitrification of cryo samples: protein structure and beam-induced motion. *Acta Crystallographica Section D*, 78(7):883–889, Jul 2022. .
37. G. McMullan, K.R Vinothkumar, and R. Henderson. Thon rings from amorphous ice and implications of beam-induced Brownian motion in single particle electron cryo-microscopy. *Ultramicroscopy*, 158:26–32, 2015.
